# Deep Evolutionary Forecasting identifies highly-mutated SARS-CoV-2 variants via functional sequence-landscape enumeration

**DOI:** 10.21203/rs.3.rs-2248327/v1

**Published:** 2022-12-02

**Authors:** Mireia Solà Colom, Jelena Vucinic, Jared Adolf-Bryfogle, James W. Bowman, Sébastien Verel, Isabelle Moczygemba, Thomas Schiex, David Simoncini, Christopher D. Bahl

**Affiliations:** Institute for Protein Innovation, Boston, Massachusetts, 02115, USA; Division of Hematology/Oncology, Boston Children’s Hospital, Harvard Medical School, Boston, Massachusetts, 02115, USA; AI Proteins, Boston, Massachusetts, 02215, USA; Université Fédérale de Toulouse, ANITI, IRIT-CNRS UMR 5505, Université Toulouse I Capitole, 31000 Toulouse, France; Institute for Protein Innovation, Boston, Massachusetts, 02115, USA; Division of Hematology/Oncology, Boston Children’s Hospital, Harvard Medical School, Boston, Massachusetts, 02115, USA; Institute for Protein Innovation, Boston, Massachusetts, 02115, USA; Division of Hematology/Oncology, Boston Children’s Hospital, Harvard Medical School, Boston, Massachusetts, 02115, USA; AI Proteins, Boston, Massachusetts, 02215, USA; Université Littoral Côte d’Opale, UR 4491, LISIC, F-62100 Calais, France; Institute for Protein Innovation, Boston, Massachusetts, 02115, USA; Division of Hematology/Oncology, Boston Children’s Hospital, Harvard Medical School, Boston, Massachusetts, 02115, USA; AI Proteins, Boston, Massachusetts, 02215, USA; Université Fédérale de Toulouse, ANITI, INRAE-UR 875, 31000 Toulouse, France; Université Fédérale de Toulouse, ANITI, IRIT-CNRS UMR 5505, Université Toulouse I Capitole, 31000 Toulouse, France; Institute for Protein Innovation, Boston, Massachusetts, 02115, USA; Division of Hematology/Oncology, Boston Children’s Hospital, Harvard Medical School, Boston, Massachusetts, 02115, USA; AI Proteins, Boston, Massachusetts, 02215, USA

## Abstract

Host-pathogen interactions drive an evolutionary game of cat-and-mouse between a pathogen’s protein virulence factors, the host’s adaptive immune system, and therapeutics targeting the pathogen. There is an urgent need for treatments and prophylactics that remain effective as a pathogen evolves, and the ability to predict pathogen evolution is a longstanding challenge. Therefore, a common strategy has been to target conserved epitopes, but strong selective pressures can drive pathogens to evolve resistance nonetheless. Here, we report a novel, generally-applicable approach called Deep Evolutionary Forecasting that predicts protein evolution using artificial intelligence and molecular modeling. The first step is to perform a complete enumeration of the functional sequence landscape in silico for a target protein. Then, we construct a graph where the edges between sequence variants are weighted by evolutionary probability. Protein evolution is forecasted by traversing this graph. We chose the SARS-CoV-2 receptor binding domain (RBD) as a model system because highly-mutated viral variants have continued to emerge that escape available therapeutics and vaccines. The RBD variants that we forecasted carry up to 11 concurrent amino acid substitutions at the host receptor binding site. Pseudoviruses harboring forecasted RBDs are active and escape binding and neutralization by FDA-approved monoclonal antibody therapeutics. We identified bottlenecks in the evolutionary landscape of SARS-CoV-2 that are promising targets for therapeutics that preempt evolution.

All current vaccines and therapeutics for infectious diseases work by targeting pathogen virulence factors that already exist. For example, our response to endemic pathogens like influenza is to continuously develop and administer seasonal vaccines to protect against newly emerged variants^1^. Similarly, it is all too common for monoclonal antibody treatments for infectious disease to decline in efficacy as pathogens inevitably evolve resistance^6^. Much like we do with weather, humanity needs the ability to forecast pathogen evolution and predict amino acid changes long before they occur. We call this “deep evolutionary forecasting”. This capability will enable the design of vaccines and therapeutics which are able to retain their efficacy against novel variants and prevent them from spreading.

The novel betacoronavirus SARS-CoV-2 emerged in late 2019 and caused a global pandemic that has resulted in more than 6 million deaths thus far^7^. While effective vaccines^8^ and therapeutics^9^ were developed with unprecedented speed, this wasn’t sufficient to keep up with the pace of viral evolution. New strains quickly emerged that were able to infect vaccinated persons and escape neutralizing monoclonal antibody treatments^10–12^. Inevitably, SARS-CoV-2 will continue to evolve^13^.

To infect human cells, the SARS-CoV-2 spike (S) protein homotrimer must bind to its receptor, the angiotensin-converting enzyme 2 (ACE2) homodimer, on the host cell surface^14^. The receptor binding domain (RBD) on the S protein contains all amino acids which directly contact ACE2, and blocking the ACE2:RBD interaction prevents the virus from infecting cells^15–17^. Thus, the RBD is the target of most known neutralizing antibodies. We chose to focus our studies on the ACE2 binding site of the RBD from the L strain, which is the first strain of SARS-CoV-2, originally identified in December of 2019.

## Enumeration of functional sequence space

**Figure 1:**
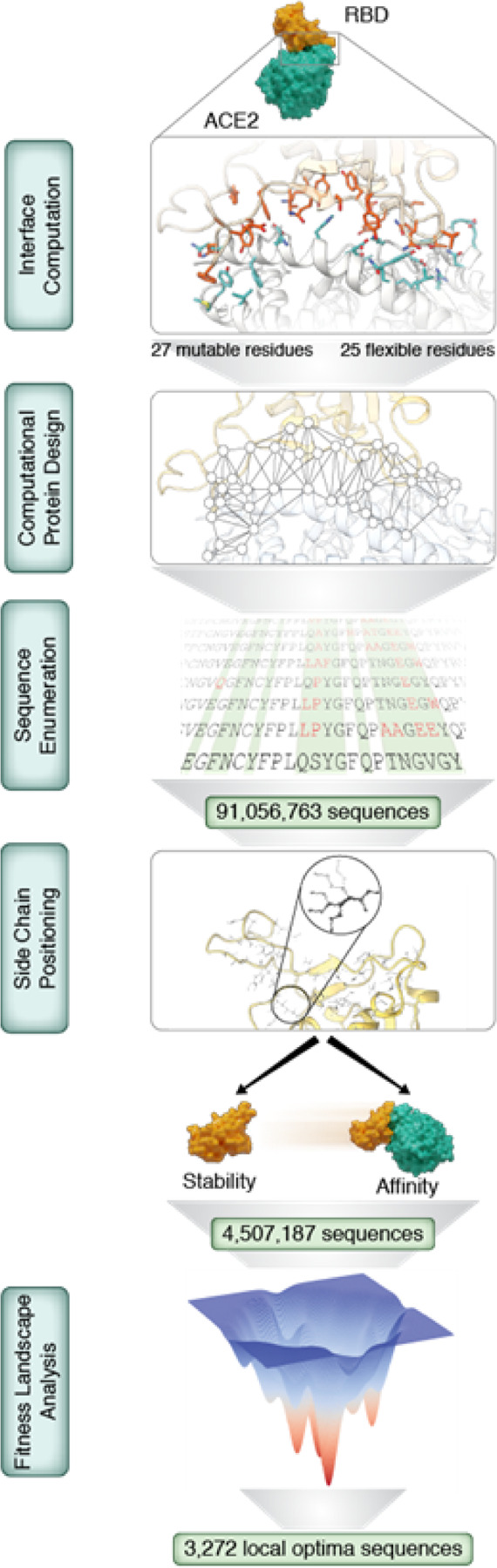
Computational workflow for deep evolutionary forecasting. **Interface computation:** Interface residues are computed on the ACE2/L strain RBD complex. **Computational Protein Design:** the global minimum energy conformation is computed. **Sequence enumeration:** all sequences within a 8 kcal/mol threshold are enumerated. **Side chain positioning:** the energy of all sequences enumerated on the complex form is computed on the RBD apo form. Next, filters are applied in order to only keep sequences with stable apo conformation and good affinity towards ACE2. **Fitness Landscape Analysis:** the ∆∆G mutational fitness landscape is analyzed and 3,272 local optima sequences are retained.

The first step in forecasting pathogen evolution is to understand the impact that every combination of mutations will have on the target protein. We began by using Rosetta to identify the interacting residues at the ACE2:RBD interface from the first high-resolution structure of this complex^15^ (Fig. 1). We then evaluated all combinatorial mutations at the 27 interface residue positions on RBD, and simultaneously, we allowed the 25 interface residues on ACE2 to explore alternative rotamers. This defines a search space of 1.3×10^35^ sequences and more than 5×10^87^ side-chain conformations (because we evaluate all common rotamers at each residue position). This is greater than the number of atoms in the observable universe, so full enumeration of this search space is not possible using brute-force computation. Thankfully, there are many limitations that restrict what sequences can effectively be sampled by an organism. For example, successful pathogens accrue adaptive or neutral mutations in their required virulence factor proteins while constantly maintaining their ability to both fold and function. The sequences which satisfy these functional criteria represent a tiny fraction of what’s possible, and these are the sequences that we need to identify in order to forecast evolution.

Using automated reasoning artificial intelligence, we are able to place boundaries on a protein’s sequence search space and only simulate functional sequence variants. In this way, full sequence enumeration becomes a computationally-tractable NP-complete problem. To do this, we used toulbar2, a dedicated numerical automated reasoning tool that we developed based on Cost Function Networks^18,19^. With its dedicated sequence enumeration algorithm (Fig. S1), toulbar2 has impressive performance for exploring protein sequence search space^20–22^. These calculations assessed two distinct functional properties: the binding energy and folding stability (Fig 1).

**Figure 2:**
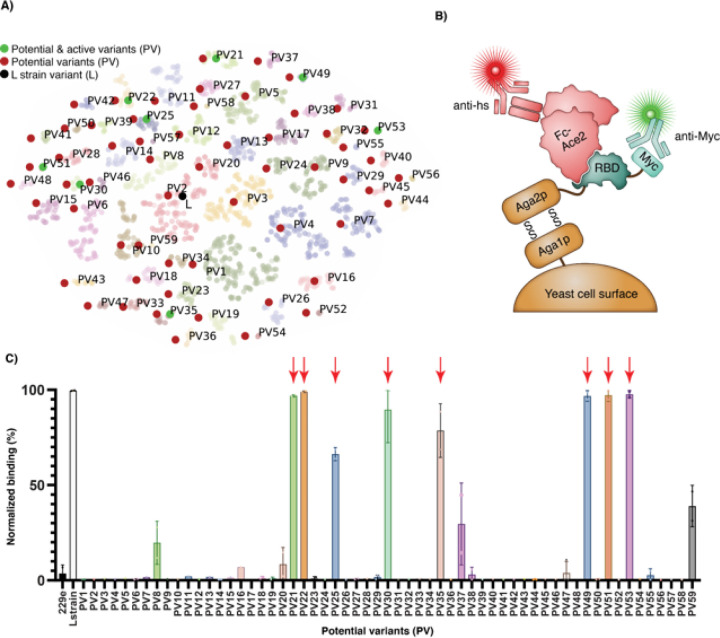
Clustering identification and binding of the 59 potential variants (PVs) to Fc-ACE2. **(A)** Spatial distribution of the 59 identified clusters on a t-distributed stochastic neighbor embedding (t-SNE) map. The L strain sequence is highlighted in black, the 59 PVs (i.e., medoids of each cluster) are highlighted in red, and the active potential variants (i.e., validated for Fc-Ace2 binding and/or infectivity) are highlighted in green. **(B)** The RBDs of the PVs were expressed on the surface of *Saccharomyces cerevisiae* cells as genetic fusions to the AGA2 surface protein. An anti-Myc antibody (Ab) and an anti-human secondary Ab labeled with different fluorophores were subsequently used to label the RBD-expressing cells and the cells bound to Fc-ACE2, respectively. **(C)** *Saccharomyces cerevisiae* cells displaying the indicated RBD PV were incubated with 40 nM Fc-ACE2. Binding to Fc-ACE2 was detected with an Alexa647-conjugated anti-human secondary Ab. The RBD variants highlighted with red arrows were chosen for further experimental characterization.

First, we assessed the binding energy between RBD and ACE2, which we approximated by the difference in kcal/mol between the bound and unbound RBD conformations, or ∆G. We used Pompd, which uses toulbar2 for sequence enumeration^23^, to compute an exhaustive list of all variant sequences capable of adopting an ACE2-bound conformation within 8 kcal/mol of the global energy minimum; this yielded over 91 million sequences (Fig. S2). Next, we evaluated the impact of these sequence changes on RBD stability, which we define as < 1 kcal/mole increase in energy (which is minimally destabilizing). For binding, we required the ∆∆G, the difference of ∆G between the L strain model and the variant model, to be positive. The intersection of these two ensembles resulted in about 4.5 million sequence variants. To reduce the size of this sequence space, we analyzed the fitness landscape defined by our 4.5 million variants, using ∆∆G as the fitness function and connecting two variants when they differ only by one mutation ^24^. In this landscape, we identified 3,272 locally optimal sequences, meaning that mutating to any of their neighbors wouldn’t improve the interface ∆∆G. Finally, we clustered the local optima by sequence similarity and identified 59 unique clusters. We visually represented the spatial distribution of these clusters on a t-distributed stochastic neighbor embedding map (Fig. 2A). We selected the medoid of each cluster for characterization in the wet laboratory, and we will refer to these sequences henceforth as the Potential Variants (PVs).

The 59 PVs span a wide region of the sequence space. They each have 10 to 15 amino acid changes compared to the L strain, which in many cases is over half of all residues that interact with ACE2 (Fig. S3, Table S1). When compared to one another, the closest PVs have as few as 4 amino acid differences, and the furthest have up to 15 differences (Table S1). Interestingly, some residue positions are highly conserved across PVs, while others are highly variable. Notably, aromatic residues in the L strain are more likely to be conserved between our PVs, and this is consistent with the variability observed in the GISAID database of clinical isolates (Fig. S4). Overall, our PVs exhibit higher sequence entropy than currently identified clinical isolates (Fig. S5).

## Potential variant RBDs bind ACE2

We next sought to assess the fidelity of our *in silico* predictions using *in vitro* experiments. We obtained synthetic genes encoding the 59 PVs and expressed them from *Saccharomyces cerevisiae* as fusions to AGA2 on the cell surface. This system enabled us to rapidly evaluate all of the PV RBDs for the ability to bind their receptor ACE2 using yeast display and fluorescence activated cell sorting (Fig. 2B). We used the RBD from the L strain as the positive control, and the RBD from human coronavirus 229E, which binds to a different receptor, as a negative control^25^. ACE2 was expressed, purified and used as an Fc fusion, as this construct recapitulates the dimer that this protein forms endogenously.

From the 59 PVs, 11 showed statistically significant binding at 40 nM Fc-ACE2 (Fig. 2C), which is the K_D_ for the soluble RBD of the L strain, and 8 of these PV RBDs bound at levels comparable to the L strain. Some PVs exhibited binding at Fc-ACE2 concentrations as low as 1 nM (fig. S6). This result was encouraging, especially considering the high amount of protein sequence changes.

**Figure 3:**
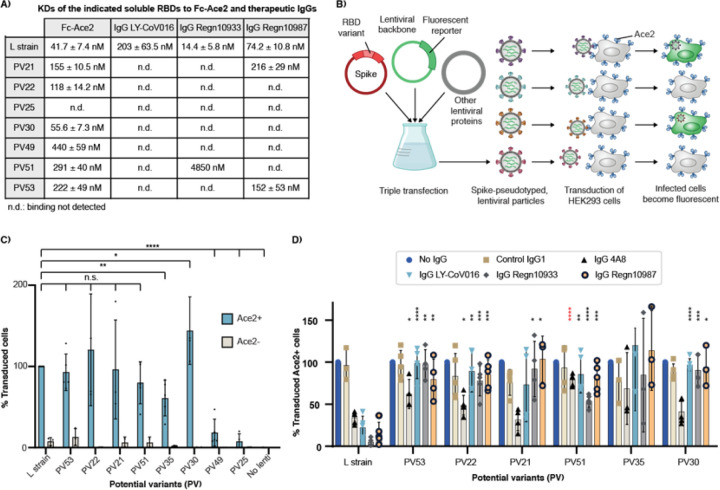
Binding affinities and infectivities of Ace2-expressing cells of the PVs and the L strain. **(A)** Reported KDs of the indicated RBD variants to Fc-ACE2 and the neutralizing antibodies as analyzed by biolayer interferometry (BLI). Binding interactions that are too weak for quantitation are labeled not determined (n.d.). BLI sensograms and fitted curves are shown in Fig. S7. **(B)** Fluorescent lentivirus pseudotyped with the SARS-CoV-2 S protein containing the different RBD potential variants (PV) were used to transduce ACE2-expressing HEK293 cells. In this setup, cell entry is dependent on ACE2 expression and cell fluorescence can be measured as a read-out for lentivirus transduction. **(C)** Equal amounts of lentivirus expressing the different PVs were used to transduce ACE2-expressing (ACE2+) and wild-type (ACE2-) HEK293 cells. Cell fluorescence was measured by flow cytometry and normalized by the % of cells transduced by lentivirus pseudotyped with the L strain. Data from 3 biological replicates is shown. **(D)** To assess the neutralization capacity of the indicated therapeutic antibodies, 10 x excess of the estimated IC50 concentrations were pre-incubated with the different pseudotyped lentivirus variants before adding them to ACE2+ cells. Fluorescence values are normalized by the no IgG control. P-values < 0.05 as compared to the L strain RBD lentiviral particles are shown: n.s.: no significant. *, **, ***, ****: p values < 0.05, 0.005, 0.0005, 0.00005, respectively.

We obtained mammalian expression constructs for the 8 PV RBDs that showed best binding, as well as the L strain and 229E. RBDs from seven PVs could be purified with yields similar to those of the L strain. PV35 was not able to be expressed in this system. We used biolayer interferometry (BLI) to measure the binding affinity of each RBD to Fc-ACE2. Binding to the L strain RBD was concordant with previously reported affinities^26,27^, and the PV RBDs bound to Fc-ACE2 with varying affinities (Fig. 3A, Fig. S7). PV30 bound with the tightest affinity, which was similar to that of the L strain. No binding was detectable for PV25.

## Potential variant pseudoviruses

For deep evolutionary forecasting to be useful, it must be capable of predicting protein sequences of novel infectious viruses. We used non-infectious pseudovirus particles to model viral cell entry, as this approach is safe and has been demonstrated to reliably recapitulate this stage of the viral life cycle^28^. We produced SARS-CoV-2 S-pseudotyped lentiviral particles in which the different PV RBDs replace the L strain RBD (Fig. 3B). Following purification, we quantified the pseudovirus particles using real-time PCR (fig. S8), and equal amounts were used to transduce both ACE2-negative (ACE2-) and ACE2-expressing (ACE2+) HEK293 cells. Not surprisingly, the pseudotyped viral particles expressing the six PVs that recognized Fc-ACE2 in BLI experiments were also able to transduce ACE2+ cells. The PV with the lowest Fc-ACE2 affinity, PV49, showed the least efficient transduction (fig. 3C). Interestingly, PV35, which could not be expressed as a soluble RBD (see above), was able to efficiently transduce ACE2+ cells, suggesting that it can be stably expressed in the context of the S protein.

## Neutralizing antibody escape

Next, we assessed how efficacious the current FDA-approved neutralizing monoclonal antibody therapeutics would be for treating infection by a forecasted PV. We expressed and purified the two antibodies from the Regeneron cocktail (Regn-10933 and Regn-10987^29,30^), as well as Eli Lilly Ly-CoV016^30^, which recognizes an overlapping but not identical epitope on the RBD^31^. We used BLI to measure the binding of these antibodies to purified RBD. While all three antibodies bound the L strain RBD, the PVs exhibited substantially diminished binding. All tested PVs escape Ly-CoV016, while only PV51 is recognized by the Regn10933 antibody at a very low affinity. In addition, only two PVs (PV21 and PV53) are recognized by the Regn10987 antibody, but also at a decreased affinity compared to the L strain RBD (fig. 3A). Finally, we evaluated the neutralization capacity of the therapeutic antibodies on the pseudovirus particles expressing the different PVs. In addition to Regn10933, Regn10987 and Ly-CoV016, we included the neutralizing antibody 4A8, which neutralizes SARS-CoV-2 infection by binding to the NTD of the S protein^32^. The neutralization capacity of the Regn10933, Regn10987 and Ly-COV016 antibodies decreased for pseudovirus particles expressing all PVs in comparison to those expressing the RBD of the L strain (Fig 3D). In contrast, the 4A8 antibody was still able to neutralize most of the PVs, as expected. Together, these data indicate that forecasted receptor binding sites on viral cell entry proteins are capable of forming infectious virions that evade extant therapeutics.

## Forecasting evolutionary trajectories

**Figure 4:**
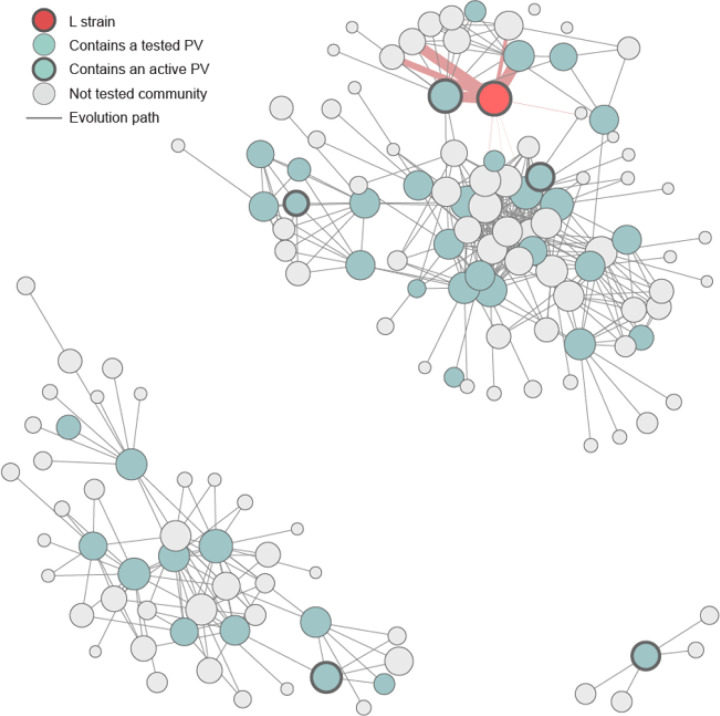
Sequence community graph. Each node represents a community of sequences. The red node represents the community that contains the L strain variant and blue nodes represent communities that contain tested PVs. Nodes with thick circles represent communities that contain active PVs. Thickness of red edges shows how connected the L strain community is.

The final and most important step in deep evolutionary forecasting is to identify regions of protein sequence space to focus on for therapeutic and vaccine development. To do this, we need to understand the possible evolutionary trajectories through the accessible sequence landscape, *i.e*., the series of amino acid substitutions that a protein may accrue over time. When a protein’s function is absolutely required for an organism’s fitness, as is the case with viral spike proteins, it is reasonable to assume that non-functional sequences are highly unlikely to propagate. Furthermore, an important consequence of DNA encoding is that some amino acid substitutions are more likely to occur than others^33^. Based on these assumptions, we constructed a graph of the sequence fitness landscape where edges between nodes are weighted by mutational probability (Fig. 4). We looked for the most probable mutational paths from the L strain to several PVs in the ∆∆G fitness landscape. For all of them, we found evolutionary paths with lengths varying from 7 to 16 single mutations, in which all evolutionary intermediates remain in the functional sequence landscape (Table S3). To have a higher-order view of the viral evolutionary graph topology, we computed communities, which are subsets of tightly connected variants. Such communities are separated from the others by a few edges that must be traversed in order for evolution to reach them. For the SARS-CoV-2 RBD, the resulting community graph shows three isolated “sequence islands.” Thus, it is highly unlikely that the virus will be able to evolve across the gap between islands. Only four communities are strongly connected to the L strain community (Fig 4). One of our active PVs (PV30) lies in the L strain community and another (PV51) belongs to a neighboring community. Viral evolution could be confined by designing vaccines that protect against the most probable and infectious variants linking the L strain community to these neighboring communities.

## Limitations and future outlook

The work presented here represents an important first step, and methods for deep evolutionary forecasting require further improvement. First, the false-positive prediction rate must improve, as many PVs did not bind to ACE2 in our experiments. Second, the false-negative rate must also improve. We performed these studies before the emergence of the Omicron variant, and retrospective analysis revealed that 4 out of the 7 sampled Omicron mutations did not appear in our enumerations, including Q498R and N501Y. The Q498R and N501Y mutations have a significantly higher energy than their wild-type counterparts in our calculations, likely due to steric clashes (Fig. S11). Proteins are not static molecules, and solving these challenges will likely require accounting for protein dynamics during enumeration, as well as enumerating a larger number of residues at one time to account for long-range epistatic effects that are not directly part of the functional site.

To our knowledge, this study is the first demonstration that deep evolutionary forecasting is possible. In contrast to other approaches^34,35^, deep evolutionary forecasting enumerates the entire sequence space of a functional protein site. Longitudinal genetic sequencing is not required, so deep evolutionary forecasting can be used immediately after a novel pathogen is discovered. A unique and important advantage of this approach is its ability to predict epistatic effects (Table S5). This enabled us to identify several highly-mutated PVs that support viral fitness, yet are evolutionarily isolated by non-functional sequences on an inaccessible sequence island. This indicates that myriad functional protein sequences exist that are largely inaccessible to life via Darwinian evolution.

Deep evolutionary forecasting paves the way for computationally designed vaccines that need to be updated less frequently, and may allow the eradication of all human viruses, including ones which evolve too rapidly for current vaccine technologies to contain. The PV sequences we report in this study can immediately be incorporated into vaccines for COVID-19 and should improve their durability to viral evolution.

## Methods

### Protein models preparation

The crystal structure of the SARS-CoV-2 spike receptor-binding domain (RBD) bound to the ACE2 receptor was retrieved from the Protein Databank (pdb code 6M0J) and used as a starting point for protein models preparation. Two protein models were derived from this crystal structure : the RBD/ACE2 complex form and the RBD unbound form. Both structures were relaxed 100 times using rosetta modeling suite version 3.12 ^36,37^ and the lowest scoring models were kept. The relaxations were made with coordinate constraints ensuring that the models do not deviate by more than 0.15 Angstroms from the initial crystal structure ^38^. Additional flags were set in order to account for glycosylated amino acid residues.

After relaxation, the RBD/ACE2 interface residues were computed on the complex form using Rosetta scripts and the InterfaceByVector residues selector with default settings. 27 and 25 residues were selected respectively on the RBD and ACE2 side.

### Computational protein design and exhaustive sequence enumeration

Computational protein design and exhaustive suboptimal sequence enumeration tasks were performed on the RBD/ACE2 complex protein model using POMPd ^23^. POMPd relies on PyRosetta to compute energy matrices. PyRosetta version r245 was used in this project.

Mutable, flexible and rigid residues were defined as follows: the 27 interface residues on the RBD side were mutable, the 25 interface residues on the ACE2 side were flexible and all other residues were rigid. Mutable residues are allowed to mutate to any of the 20 natural amino acid types, flexible residues can reorient their side chain without changing their amino acid type and rigid residues are completely frozen. The Dunbrack 2010 rotamer library ^39^ was used to define the conformational search space. The basic level of rotamer discretization was used, no extra rotamers were added on chi angles. The Rosetta genpot energy function was used, and additional flags were set in order to account for glycosylated amino acid residues. For design, POMPd calls toulbar2 with flags “-dee: -hbfs: -m -A -s --cpd“. For enumeration the additional flags “--scpbranch -a -ub <E_max_>” are added. All calculations were performed on the CALMIP high performance computing cluster, using Intel Skylake 6140 2.3 Ghz CPUs.

### Calculations on the RBD/ACE2 complex form

A side chain positioning task was performed on the L strain structure in order to compute its optimal energy, which was determined to be −1694.57 kcal/mol. The Global Minimum Energy Conformation (GMEC), the optimal sequence using our settings, was computed. The GMEC was found to have an energy of −1704.42 kcal/mol. An exhaustive enumeration of suboptimal sequences was then performed using an energy threshold of 8 kcal/mol above the GMEC. 91,056,763 different sequences satisfying the threshold were identified. The energy threshold of 8 kcal/mol was determined to be the maximum value for which results could be obtained in one day time with the computational resources used approximately 400 gigabytes of RAM, and given that the number of sequences grows exponentially with the size of the enumeration threshold (Fig S2). The L strain sequence is not present in the enumerated sequences, it is located at 9.85 kcal/mol from the global optimum.

### Calculations on the RBD unbound form

A side chain positioning task was performed on the L strain RBD unbound form using toulbar2. Its optimal energy, using our settings, was determined to be −386.74 kcal/mol.

For each one of the 91 million sequences found in the enumeration, the RBD unbound form energy was also computed by solving 91 million NP-complete side chain positioning problems to optimality. The 27 interface residues were defined as flexible and all other residues were kept rigid. The computation was performed using a parallel implementation MPI-based variant of toulbar2. It was completed in less than 2 days on 200 CPU cores.

### ∆∆G fitness landscape

∆∆G values were computed as $\Delta \Delta \text{G}=\Delta {\text{G}}_{\text{mut}}-\Delta {\text{G}}_{\text{wt}}=\left({{\text{E}}^{\text{complex}}}_{\text{mut}}-{{\text{E}}^{\text{apo}}}_{\text{mut}}\right)-\left({{\text{E}}^{\text{complex}}}_{\text{wt}}-{{\text{E}}^{\text{apo}}}_{\text{wt}}\right)$ where ${{\text{E}}^{\text{complex}}}_{\text{mut}}$ and ${{\text{E}}^{\text{apo}}}_{\text{mut}}$ are the energies of each mutant in the enumeration, respectively in complex and apo forms, ${{\text{E}}^{\text{complex}}}_{\text{wt}}$ and ${{\text{E}}^{\text{apo}}}_{\text{wt}}$ are the energies of the L strain respectively in complex and apo forms. Prior to computing the fitness landscape, the 91 million sequences were filtered in order to retain only sequences having a sufficiently stable RBD unbound form, with energy less than 1 kcal/mol worse than the L strain and a negative ∆∆G energy (with increased predicted affinity towards ACE2). The remaining 6,390,176 sequences were further filtered in order to remove all mutants exhibiting unpaired cysteine mutations. The final set includes 4,507,187 different sequences. The fitness landscape was computed on the final set of sequences, using a Hamming distance of 1 as neighborhood and ∆∆G energy as the fitness function. We could include the L strain variant in the fitness landscape since it is a neighbor of two sequences.

### Local optima cluster representatives calculation

The fitness landscape contained 3,272 local optima, which were clustered with mmseqs using a sequence identity threshold of 80%:

mmseqs easy-cluster in_fasta out_clusters tmp --min-seq-id 0.8

The clustering produced 59 clusters. Each cluster medoid was then identified, and the 59 corresponding sequences were selected for experimental analysis. A sequence logo representing all local optima was computed using Weblogo ^40^.

### Most probable paths from the L strain to active potential variants

We calculated shortest paths between the L strain and active potential variants in the ∆∆G fitness landscape graph in which edges were weighted by mutational probabilities. We used DNA level mutation rates estimated by maximum likelihood using MEGA ^41^ with the General Time Reversible model (best fit under AIC and BIC regularization) extracted from ^42^ on coronavirus genomic sequences. From this, we computed a transition probability matrix at the DNA level using matrix exponentiation, with a time parameter adjusted to get an expected number of DNA mutations of around one mutation over the designed RBD region (with 27 residues). Matrix exponentiation was computed using the Pade approximation available in Python scikit as the scipy.linalg .expm function. The resulting transition matrix gives access to transition probabilities P(M[W) that a given DNA base W (in the L strain) will mutate to a base M in the next time-slice. To compute the amino acid level mutation rates induced by this DNA transition matrix, we first computed a codon to codon transition probability matrix, assuming independent identically distributed mutation rates given by the previous matrix. For a given amino acid A, let lc(A) be the set of synonymous codons representing amino acid A, the a priori probability that a given codon c in lc(A) is used to represent A is simply $\text{f}\left(\text{c}\right)=\text{r}\left(\text{c}\right)/\left|\text{lc}\left(\text{A}\right)\right|$ where r(c) is the Relative Synonymous Codon Usage (RSCU) of the synonymous codon c, as computed for SARS-CoV-2 coronavirus ^43^. The probability for an amino acid W, represented by a latent codon variable, to mutate in an amino acid M (represented by any of its synonymous codon) is then P(M|W) = Sum_W in lc(W) f(cW) Sum_cM in lc(M) P(cM|cW). The -log of this transition probability matrix was used to weight the edges connecting two sequences in our variant landscape. The weight of a minimum cost path between two variants then defines a most likely path from the source variant to the target variant. Dijkstra’s algorithm was used to compute the shortest paths from the L strain to active potential variants.

### Sequence community graph

The community graph was calculated from 4,507,188 sequences (including L strain variant). It was partitioned using the Leiden algorithm and modularity as a quality measure. Each node in the graph represents a community of sequences. Edges between the nodes were weighted with mutational probabilities described previously. The size of the nodes is proportional to the log of the size of communities. The thickness of edges connecting the L strain community is proportional to the log sum of all L strain community outgoing edges weights. All nodes with a degree smaller than 3 were removed. Self edges were removed and the graph was made undirected. The Leiden algorithm was run for 50 iterations and appears to be stable with a modularity of 0,93. Calculations were done using python leidenalg library. The partition was computed with the following command:

la.find_partition(g, weights = ‘weights’, partition_type=la.ModularityVertexPartition, n_iterations=50)

### Theoretical affinity of antibodies towards L strain RBD and potential variants.

Three different antibodies in complex with L strain RBD were used for ∆∆G calculations (pdb codes : 6XDG and 7C01). These complexes were relaxed 100 times using rosetta modeling suite version 3.12 (genpot scoring function) and the lowest scoring models were kept. Coordinate constraints were set in order to ensure that the models do not deviate by more than 0.15 Angstroms from the initial crystal structure. Additional flags were set in order to account for glycosylated amino acid residues. The energy of each of these complexes was calculated.

The energies of the potential variants in complex with antibodies were obtained by side chain positioning calculations.

### Identification of matching natural RBD sequences

We downloaded natural spike protein sequences from GISAID (https://www.gisaid.org/), using the spikeprot0125.tar archive containing 7,352,708 and only kept the 7,241,769 sequences with length above 1620, representing putative full-length sequences (possibly containing wildcard characters ‘X’). Identifying the subset of our 4,507,187 RBD motifs that appears in the database would require more than 20,000 billions pairwise alignments. Suspecting that only few of these would appear in the natural diversity, we exploited the gap structure of the motifs to look for matches of partial dense sub-motifs in the GISAID set. The submotif defined by the 18 last residues of our RBD motif contains only short gaps (the longest being 7 residues long). Our 4,507,187 RBD motifs contain only 152,487 different combinations of these 18 residues. We sorted this set alphabetically and divided it into 50 subsets. Exploiting the fact that any finite language is regular, we built a regular expression containing the disjunction of the motifs appearing in it and compiled it to a Deterministic Finite State Automata (DFA). By bringing similar motifs closer together, sorting before splitting increases the likelihood that the automata size will be small. Search was performed only in the subregion of the full sequences starting at position 451 and ending at position 519. DFA compilation and search was performed using Google’s re2 library (https://github.com/google/re2), as available in the Python API pyre2 (https://pypi.org/project/pyre2/). The sets of RBD motifs that yielded no hit were discarded and the same process of division in subsets, disjunction, automata compilation and search repeated recursively until singleton sequences with hits in GISAID were identified. We then selected full RBDs containing one of these 18-residue motifs with GISAID-hits and repeated the same search process. We found no occurrence of these in the GISAID sequences. We therefore repeated the same overall process, allowing this time for precisely one mismatch. With this added matching flexibility, our set of designed RBDs had 4,905,597 hits in GISAID (67.7%), covering the L strain, Delta and Lambda VOCs (Variants Of Concern), from a total of 51 designed RBDs (see Table S5-GISAID matches). With one extra mismatch allowed, the Alpha, Kappa, Eta and Iota VOCs are also covered.

### Extraction of 27-residues RBD motifs from GISAID

From all sequences of the spike protein in the spikeprot0125.tar archive, we kept the 7,241,769 sequences with length above 1,620, representing putative full-length sequences (possibly containing wildcard characters ‘X’). From each sequence, we extracted the region from position 454 to 555 that was expected to contain the RBD design region (positions 404 to 505 in the L strain). We removed all sequences containing ‘X’ and removed duplicate sequences. A multiple sequence alignment was computed with mafft, using the L strain protein S sequence as a reference, preserving length (using mafft flags --6merpair --thread -1 --keeplength --addfragments). The 27 residues of interest were extracted from each sequence, resulting in a set of 826 different 27-mers. All sequences with remaining gaps were removed, resulting in a set of 774 unique gapless 27-mers. A sequence logo was computed from this set using Weblogo.

### 2D map of ∆∆G local minima landscape

We projected the ∆∆G local minima landscape on a 2D map using t-distributed stochastic neighbor embedding (t-SNE) as implemented in the python sk-learn package. We defined a customized distance metric in order to ensure that local minima clusters computed with mmseqs2 are correctly identified by t-SNE:

$$\text{d}\left({\text{s}}_{1},{\text{s}}_{2}\right)=\text{Hamming}\left({\text{s}}_{1},{\text{s}}_{2}\right)+\text{λ}.\text{same}\_\text{cluster}\left({\text{s}}_{1},{\text{s}}_{2}\right)$$

Where Hamming is the Hamming distance between two sequences (*i.e.*, number of mutations), same_cluster is a function which returns 1 if two sequences belong to the same cluster and 0 otherwise, and λ is a control parameter (λ=20 in our calculations). The t-SNE algorithm was run for 1000 iterations with a learning rate of 50, a perplexity value of 6 and an early exaggeration value of 12.

### Sequence entropy

The sequence entropy of the 27 residues of the RBD interface were computed on the 3272 local optima of the ∆∆G fitness landscape, as well as on the 774 unique sequences extracted from GISAID. The Shannon entropy was calculated after normalizing the frequency of occurrence of each amino acid type at each position by the natural frequency of occurrence of amino acids as estimated in the literature ^44^.

### Yeast display experiments

DNA sequences encoding for the receptor binding domains (RBDs) of L strain SARS-CoV-2, the human coronavirus 229E and the 59 potential variants (PVs) were synthesized by Twist Bioscience. Next, they were amplified by PCR to introduce 50 nucleotides long flanking sequences complementary to the yeast display plasmid (RRID:Addgene_41522). The amplified DNA sequences and the linearized yeast display plasmid were transformed into *Saccharomyces cerevisiae* cells (Strain EBY100; ATCC) so that the yeast homologous recombination machinery ligated the DNA sequences encoding for the RBDs at the N-terminal of the Myc tag. Transformed cells were selectively grown in tryptophan-free minimal (SD-Trp-Ura) media (6.7g/L Yeast Nitrogen Base, 5.0g/L Casamino acids, 1.065 g/L MES acid, and 2% w/v dextrose) for 24 h at 30 C, with shaking. Next day, cell media was changed to SG-CAA media (2% Galactose, 0.67% Yeast Nitrogen Base, 0.5% Casamino Acids, 0.54% Sodium Phosphate Dibasic, 0.856% Sodium Phosphate Monobasic Monohydrate) to induce RBD expression for 24 h at 30 C, with shaking. Next day, induced cells were spun down for 2 mins at 2,000 x g, resuspended in HBS blocking buffer (20 mM Hepes 7.4, 150 mM NaCl, 1% (w/v) BSA) and incubated with recombinant Fc-ACE2 for 45 mins at RT, with shaking. Next, plates were washed twice with HBS blocking buffer and incubated with 1:250 diluted FITC-conjugated anti c-Myc (Immunology Consultants Lab, CMYC-45F) and Alexa647-conjugated anti-human- antibodies for 30 mins at RT, with shaking. Cells were washed twice with HBS blocking buffer and cell fluorescence was measured using an IntelliCyt high throughput flow cytometer. Cells were gated to exclude non-single cells, FITC labeling was used to select RBD-expressing cells, and Alexa647 labeling was used to quantify Fc-ACE2-binding cells. Fc-ACE2-binding is reported as percentage within the FITC+ population and was gated according to the Alexa647 signal of the positive (L strain RBD) and negative (229e RBD) controls.

### Expression and purification of recombinant soluble proteins

The DNA constructs for the RBDs of L strain SARS-CoV-2, the human coronavirus 229E and the eight PVs that showed Fc-ACE2 binding in yeast experiments were codon-optimized for mammalian cell expression, synthesized by Twist Bioscience and cloned into a mammalian expression vector as C-terminal genetic fusions to a 10xHis, a siderocalin module and a 3C protease cleavage site. The DNA construct encoding Fc-ACE2 was acquired from Addgene (#164222) and the DNA constructs encoding the four neutralizing antibodies were synthesized by Genscript. 24 μg of the respective DNA constructs were used to transfect 30 ml of suspension Expi293F (Thermo Scientific) cells at a density of 2.5E6 cells/ml in Expi293 media (Thermo Scientific) and cells were grown at 37 C in a humidified 8% CO2 incubator, with 130 rpm shaking. After 24 h, cells were feeded with 3 mM valproic acid and 0.45% glucose. After 5 days, cells were harvested for 10 mins at 1,000 x g. All RBD variants were purified using a sepharose Ni-IMAC resin (Pierce, Thermo Scientific) and eluted by 3C protease cleavage. The expressed IgG and Fc-ACE2 were purified using a protein A resin and eluted with 150 mM NaCl, 100 mM glycine (pH 2.8).

### Kinetic analyses by biolayer interferometry (BLI)

BLI experiments were performed using an Octet 8-channel system (Sartorius) using HBS blocking buffer supplemented with 0.05% (w/v) Tween-20. 30 nM Fc-ACE2 or 20 nM of the three tested therapeutic antibodies were immobilized on Octet protein A biosensors. The biosensors were dipped into wells containing purified L strain RBD, 229E or the respective PV at 2000, 1000, 500, 250, 125, 62.5 and 31.3 nM concentrations for 200 seconds, and subsequently dipped into wells containing HBS blocking buffer supplemented with 0.05% (w/v) Tween-20 for 200 seconds. Data were reference-subtracted, and curves were fitted using the GraphPad Prism association dissociation model (https://ww.graphpad.com/guides/prism/latest/curve-fitting/reg_equaton_association_then_disso.htm).

### ACE2-expressing HEK293 cell lines

All transduction and neutralization experiments were performed using an Ace2-negative (Ace2-) HEK293 cell line, and two different Ace2-positive (Ace2+) HEK 293 cell lines. One ACE2+ cell line was transiently transfected with an ACE2 plasmid (Addgene #141185) followed by three rounds of hygromycin selection. Next, ACE2 expression was validated by binding of an anti-Myc antibody (Fig S.10). The other ACE2+ cell line was created by lentiviral transduction and previously published (Wu et al. 2021). Significant differences were not observed in the results obtained with both cell lines.

### Production of spike-pseudotyped lentivirus

To produce lentiviral particles pseudotyped with the spike (S) protein, the RBDs of the chosen PVs were cloned in the context of the S protein into the Addgene # 145032 plasmid.

Subsequently, 3 μg of the corresponding spike plasmid, 12 μg of a lentiviral backbone expressing neonGreen (Addgene #162034) and 9 μg of a 2nd generation lentiviral packaging plasmid (Addgene #122600) were used for triple-transfections of 30 ml Exp293F cells. After 24 hours, cells were feeded with 3 mM valproic acid and 0.45% glucose, and lentivirus production proceeded for 4 additional days. After that, cells were pelleted for 5 mins at 1,000 x g and supernatants were filtered using 0.45 um filters (Sartorius) and stored at 4C.

The generated lentiviral particles were quantified by RT–PCR using a commercial kit (Biovision cat. No. K1471) that includes lysis buffer, reverse transcriptase, DNA polymerase and oligos annealing to the lentiviral scaffold. A standard curve was built with the provided standards and used to quantify the lentivirus amounts. Serial 10-fold dilutions of all pseudotyped lentivirus were run.

### Pseudotyped lentiviral particles transduction assays

Ace2+ and Ace2- HEK293 cells were seeded in 96 well plates at 5×10E4 cells per well in DMEM media supplemented with 10% FBS and incubated at 37 C. After 24 h, media was removed and replaced by equal amounts of all pseudovirus diluted in fresh DMEM media. After 18 hours, cell media was removed and cells were washed three times with HBS blocking buffer. Next, viral transduction was measured as neonGreen fluorescence using an IntelliCyt high throughput flow cytometer. Uninfected controls and the lentiviral particles expressing the RBD of the L strain were used to set the gates. All experiments were done in technical replicates and repeated in 3 different days, and statistical significances were calculated by unpaired t tests using the GraphPad Prism software version 9.0.

### Neutralization assays

First, we estimated the IC50 of the four purified neutralizing antibodies on pseudotyped lentiviruses carrying the L strain RBD (fig. S9), and then used a concentration corresponding to 10 x the respective IC50s for the neutralization experiments. To estimate the IC50s of the neutralizing antibodies with pseudotyped lentivirus expressing the RBD of the L strain, 10-fold dilutions of the antibodies were incubated with L strain pseudotyped lentivirus for 1 hour at 37 C and subsequently added to ACE2+ HEK293 cells. NeonGreen fluorescence was analyzed using an IntelliCyt high throughput flow cytometer and the data from 4 different experiments were used to estimate the IC50 values using the Graphpad Prism version 9.0. For the neutralization experiments, equal amounts of the lentivirus pseudotyped with the L strain or the corresponding PV variants were pre-incubated with 10 x IC50 concentrations of the 4 antibodies for 1 hour at 37 C and subsequently added to ACE2+ cells. Technical duplicates and at least 3 biological replicates of each sample were performed. Statistical significance was calculated by unpaired t tests using the GraphPad Prism software version 9.0.

## Supplementary Material

Supplement 1
