# Supplementary material for: Complete Combinatorial Mutational Enumeration of a protein functional site enables sequence-landscape mapping and identifies highly-mutated variants that retain activity
Source: Res Sq. 2023 Sep 11:rs.3.rs-2248327. Originally published 2022 Dec 2. Preprint. [Version 2] doi: 10.21203/rs.3.rs-2248327/v2 (PMC9727770; doi:10.21203/rs.3.rs-2248327/v2)
Supplement: Supplement 1 [file NIHPPrs2248327v2-supplement-1.pdf]

SUPPLEMENTARY INFORMATION

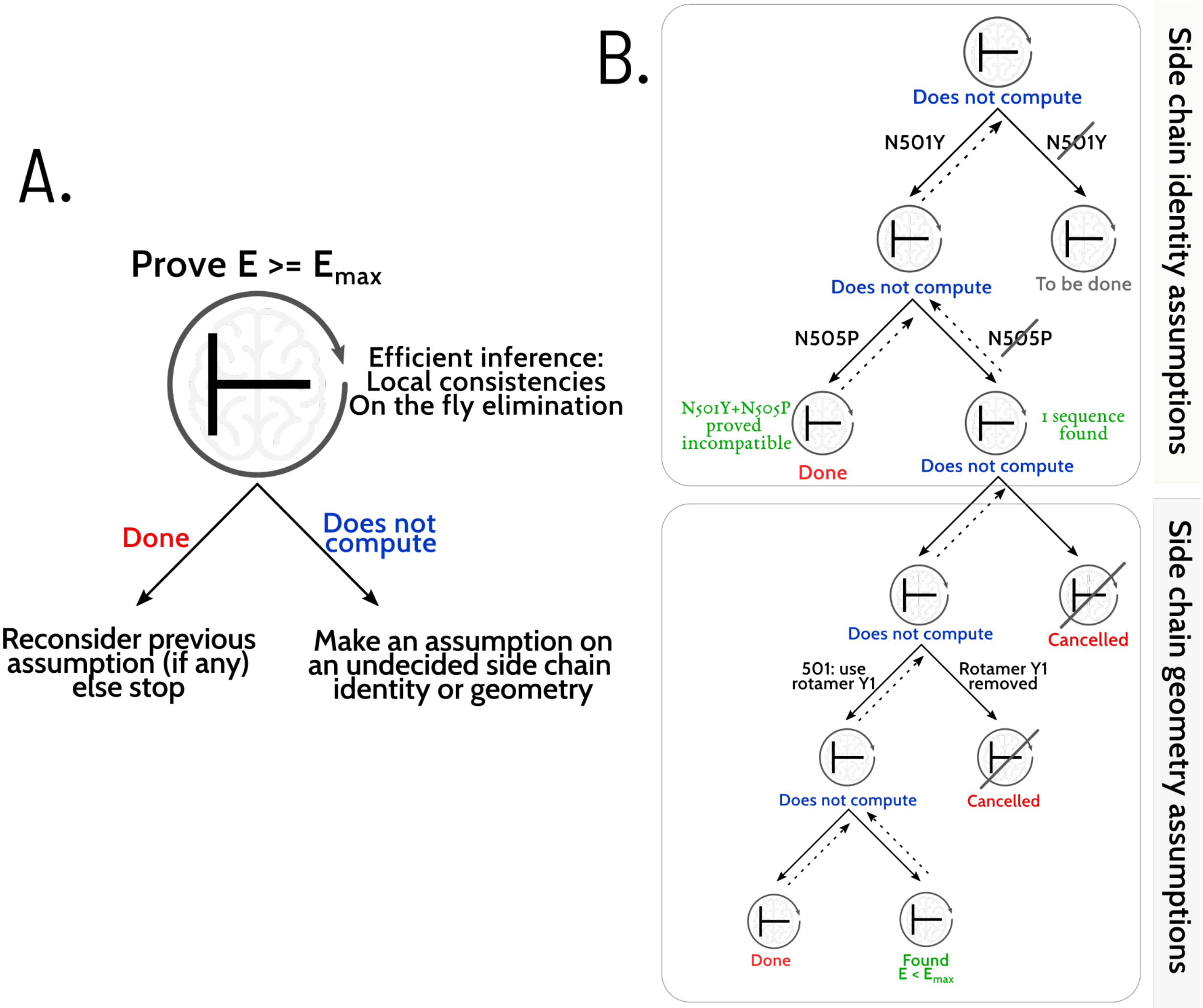

Figure S1. Toulbar2 sequence enumeration algorithm. High level description of the proof mechanisms used by toulbar2 for fast enumeration of sequences with at least one energy conformation below a threshold E_{max}.

A. Initially asked to find all sequences which can have an energy less than E_{max}, toulbar2 proceeds instead *ad absurdo*, trying to prove that all sequences must have energy above E_{max} in all their possible geometries and collects sequence counter-examples as the proof proceeds. The proof relies on efficient massive inference (symbol ⊢) by local consistencies and variable elimination [44]. Alone, these proof systems are only able to solve relatively simple problems. When the proof is out of reach, an assumption on the identity or geometry of a yet undecided side-chain is made. This makes the problem simpler and eventually solvable. If, instead, the local proof can be directly achieved, this branch of the proof is done and previous assumptions are reconsidered.

B. For sequence enumeration [9], the proof is built in two layers. In the first layer, only side-chain identities are decided. Once this is done, side-chain geometries (rotamers) are explored. As soon as a geometry of energy below E_{max} is found, a counter example (and a suitable sequence) is found and pending geometry assumptions explorations are canceled. toulbar2 therefore limits the combinatorial explosion of the protein sequence fitness landscape explored thanks to two proof pruning mechanisms: massive local inference ("Done" nodes) and counter-example based geometry pruning ("Canceled" nodes). Local inferences are also used to guide the search for counter-examples: when an assumption needs to be made, toulbar2 selects the assumption for which the last local inference was the farther away from the E_{max} target.

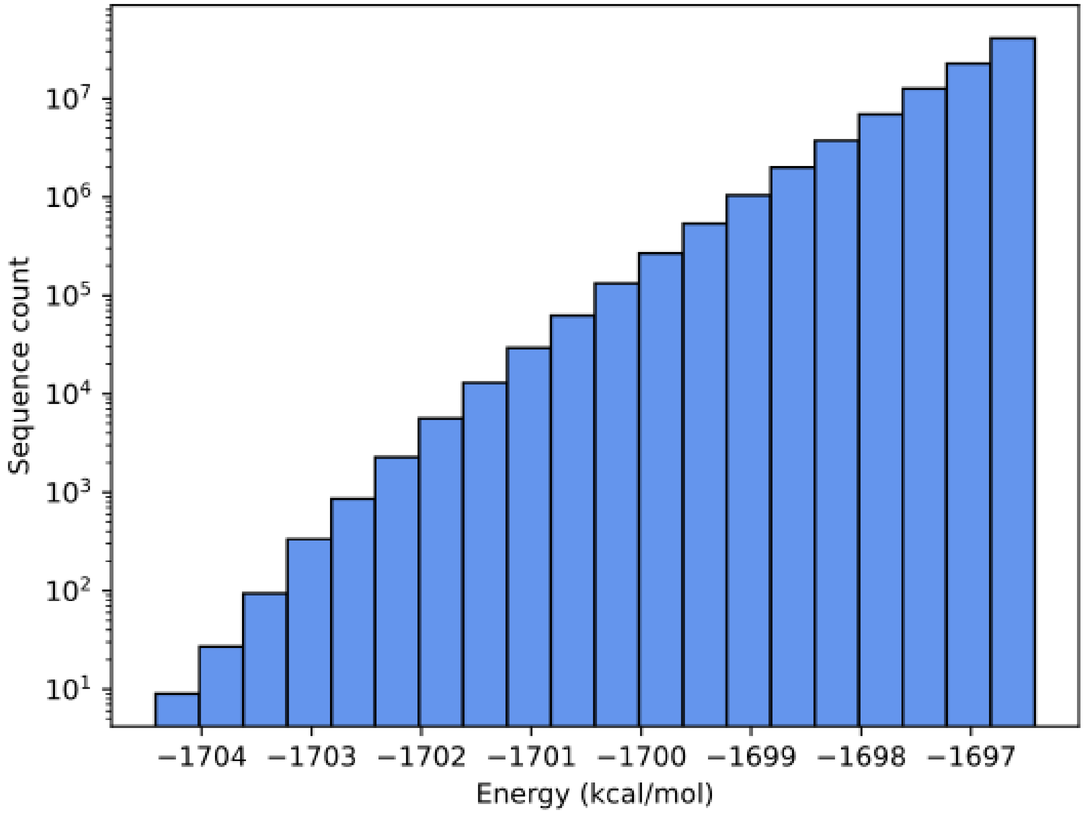

Figure S2. Energy distribution of all 91 million enumerated sequences. Non-cumulative energy distribution of sequences enumerated within 8 kcal/mol of the global minimum on the ACE2/RBD complex. The scale on the y axis is logarithmic.

```
L strain    CPFGEVFNATRFASVYAWNRKRISNCVADYSVLYNSASFSTFKCYGVSPTKLNDLCFTNVYADSFVIRGD 70
Alpha       CPFGEVFNATRFASVYAWNRKRISNCVADYSVLYNSASFSTFKCYGVSPTKLNDLCFTNVYADSFVIRGD 70
Delta       CPFGEVFNATRFASVYAWNRKRISNCVADYSVLYNSASFSTFKCYGVSPTKLNDLCFTNVYADSFVIRGD 70
Omicron     CPFDEVFNATRFASVYAWNRKRISNCVADYSVLYNLAPFFTFKCYGVSPTKLNDLCFTNVYADSFVIRGD 70
PV21        CPFGEVFNATRFASVYAWNRKRISNCVADYSVLYNSASFSTFKCYGVSPTKLNDLCFTNVYADSFVIRGG 70
PV22        CPFGEVFNATRFASVYAWNRKRISNCVADYSVLYNSASFSTFKCYGVSPTKLNDLCFTNVYADSFVIRGS 70
PV25        CPFGEVFNATRFASVYAWNRKRISNCVADYSVLYNSASFSTFKCYGVSPTKLNDLCFTNVYADSFVIRGD 70
PV30        CPFGEVFNATRFASVYAWNRKRISNCVADYSVLYNSASFSTFKCYGVSPTKLNDLCFTNVYADSFVIRGD 70
PV35        CPFGEVFNATRFASVYAWNRKRISNCVADYSVLYNSASFSTFKCYGVSPTKLNDLCFTNVYADSFVIRGD 70
PV49        CPFGEVFNATRFASVYAWNRKRISNCVADYSVLYNSASFSTFKCYGVSPTKLNDLCFTNVYADSFVIRGD 70
PV51        CPFGEVFNATRFASVYAWNRKRISNCVADYSVLYNSASFSTFKCYGVSPTKLNDLCFTNVYADSFVIRGG 70
PV53        CPFGEVFNATRFASVYAWNRKRISNCVADYSVLYNSASFSTFKCYGVSPTKLNDLCFTNVYADSFVIRGD 70

L strain    EVRQIAPGQTGKIADYNYKLPDDFTGCVIAWNSNNLDSKVGGNYNYLYRLFRKSNLKPFERDISTEIYQA 140
Alpha       EVRQIAPGQTGKIADYNYKLPDDFTGCVIAWNSNNLDSKVGGNYNYRYRLFRKSNLKPFERDISTEIYQA 140
Delta       EVRQIAPGQTGKIADYNYKLPDDFTGCVIAWNSNNLDSKVGGNYNYRYRLFRKSNLKPFERDISTEIYQA 140
Omicron     EVRQIAPGQTGNIADYNYKLPDDFTGCVIAWNSNKLDSKVSGNYNYLYRLFRKSNLKPFERDISTEIYQA 140
PV21        EVRQIAPGQTGLIADYNYKLPDDFTGCVIAWNSNNLDSKWGGNYNYLFRMFRKSNLKPFERDISTEIFQA 140
PV22        EVRQIAPGQTGVIADYNYKLPDDFTGCVIAWNSNNLDSKEGGNYNYLFRKFRKSNLKPFERDISTEIFQA 140
PV25        EVRQIAPGQTGAIADYNYKLPDDFTGCVIAWNSNNLDSKEGGNYNYLYRKFRKSNLKPFERDISTEIYQA 140
PV30        EVRQIAPGQTGLIADYNYKLPDDFTGCVIAWNSNNLDSKEGGNYNYLFRLFRKSNLKPFERDISTEIFQA 140
PV35        EVRQIAPGQTGAIADYNYKLPDDFTGCVIAWNSNNLDSKEGGNYNYLYRKFRKSNLKPFERDISTEIFQA 140
PV49        EVRQIAPGQTGWIADYNYKLPDDFTGCVIAWNSNNLDSKFGGNYNYLYRLFRKSNLKPFERDISTEIYQA 140
PV51        EVRQIAPGQTGEIADYNYKLPDDFTGCVIAWNSNNLDSKDGGNYNYLYRLFRKSNLKPFERDISTEIYQA 140
PV53        EVRQIAPGQTGLIADYNYKLPDDFTGCVIAWNSNNLDSKWGGNYNYLFRLFRKSNLKPFERDISTEIYQA 140

L strain    GSTPCNGVEGFNCYFPLQSYGFQPTNGVGYQPYRVVVLSFELLHAPA 187
Alpha       GSKPCNGVEGFNCYFPLQSYGFQPTYGVGYQPYRVVVLSFELLHAPA 187
Delta       GSKPCNGVEGFNCYFPLQSYGFQPTNGVGYQPYRVVVLSFELLHAPA 187
Omicron     GNKPCNGVAGFNCYFPLRSYSFRPTYGVGHQPYRVVVLSFELLHAPA 187
PV21        GSTPCNGVEGFNCYFPLLPYGFQPAAGEEYQPYRVVVLSFELLHAPA 187
PV22        GSTPCNGVEGFNCYFPLLPYGFQPTNGEGWQPYRVVVLSFELLHAPA 187
PV25        GSTPCNGVEGFNCYFPLLPFGFTPTAGEGWQPYRVVVLSFELLHAPA 187
PV30        GSTPCNGVQGFNCYFPLQPYGFQPTNGEGYQPYRVVVLSFELLHAPA 187
PV35        GSTPCNGVEGFNCYFPLLAFGFQPTNGEGWQPYRVVVLSFELLHAPA 187
PV49        GSTPCNGVEGFNCYFPLQAYGFQPAAGEGWQPYRVVVLSFELLHAPA 187
PV51        GSTPCNGVEGFNCYFPLQAYGFHPATGEEYQPYRVVVLSFELLHAPA 187
PV53        GSTPCNGVEGFNCYFPLVPYGFQPAAGEGYQPYRVVVLSFELLHAPA 187
```

Figure S3. Sequence alignment of the main natural variants of concern and potential variants validated in this study. Sequence alignment of the L strain, alpha, delta and omicron variants of concern together with the PVs showing the best binding to Fc-ACE2. RBD interface positions are highlighted in green, residues that differ from the L strain are shown in red. Most omicron mutations are not found in our predicted PVs. However, this is likely due to the fact that we only sampled the 27 RBD residues that contact Ace2, and other residues away from the interface might also contribute to RBD stability and indirectly to Ace2 binding. Also, the other spike domains play important roles in the viral life cycle, and viral fitness consists of a complex combination of factors that go beyond the spike. In the future, we will consider additional factors to improve our algorithm.

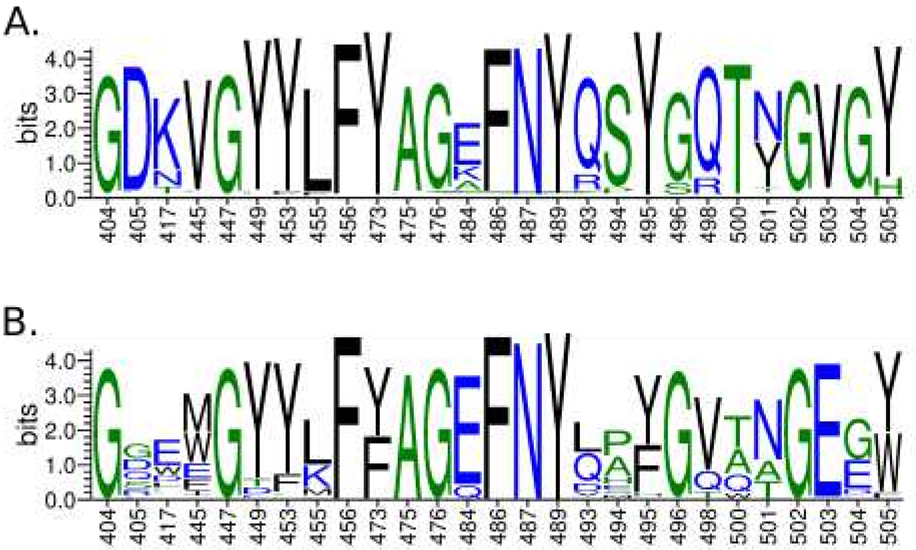

Figure S4. Sequence logos of GISAID variants and ∆∆G fitness landscape local minima. RBD interface residues sequence logo representations of 774 unique GISAID sequences (A) and ∆∆G fitness landscape local minima sequences (B).

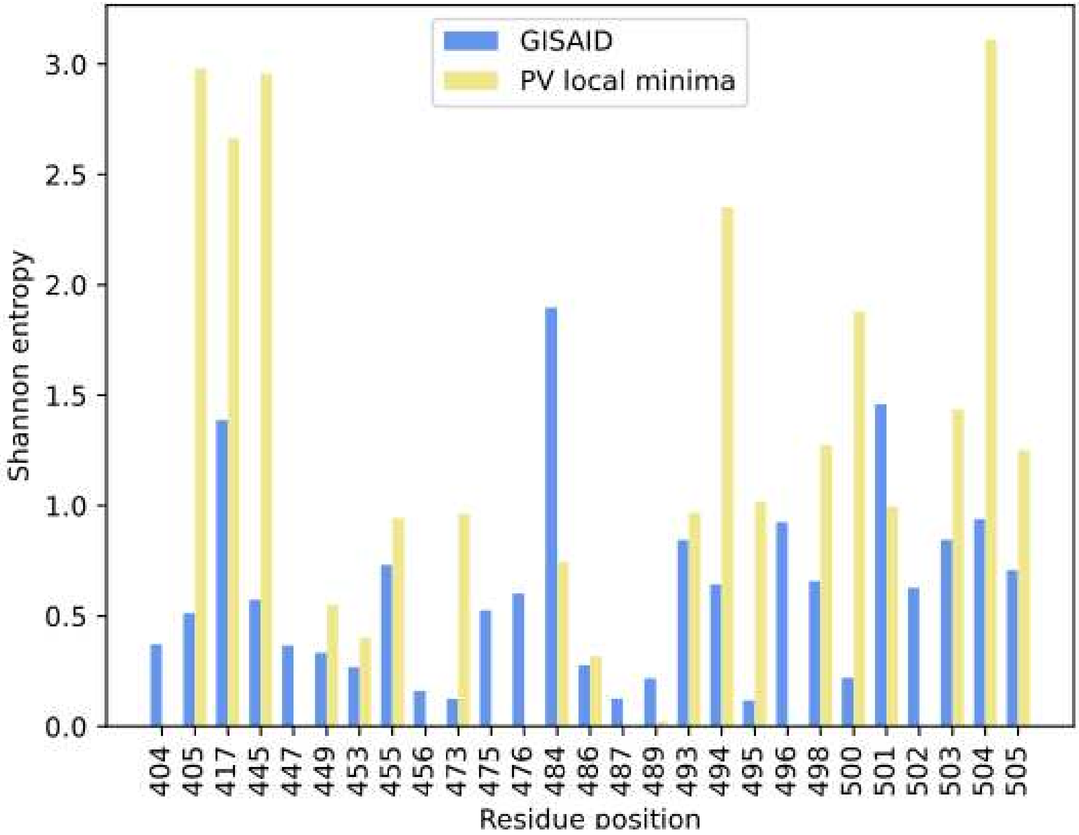

Figure S5. Shannon entropy of GISAID variants and ∆∆G fitness landscape local minima. Amino acid composition entropy of RBD interface residues for 774 unique GISAID sequences (blue) and 3272 local optima sequences (yellow).

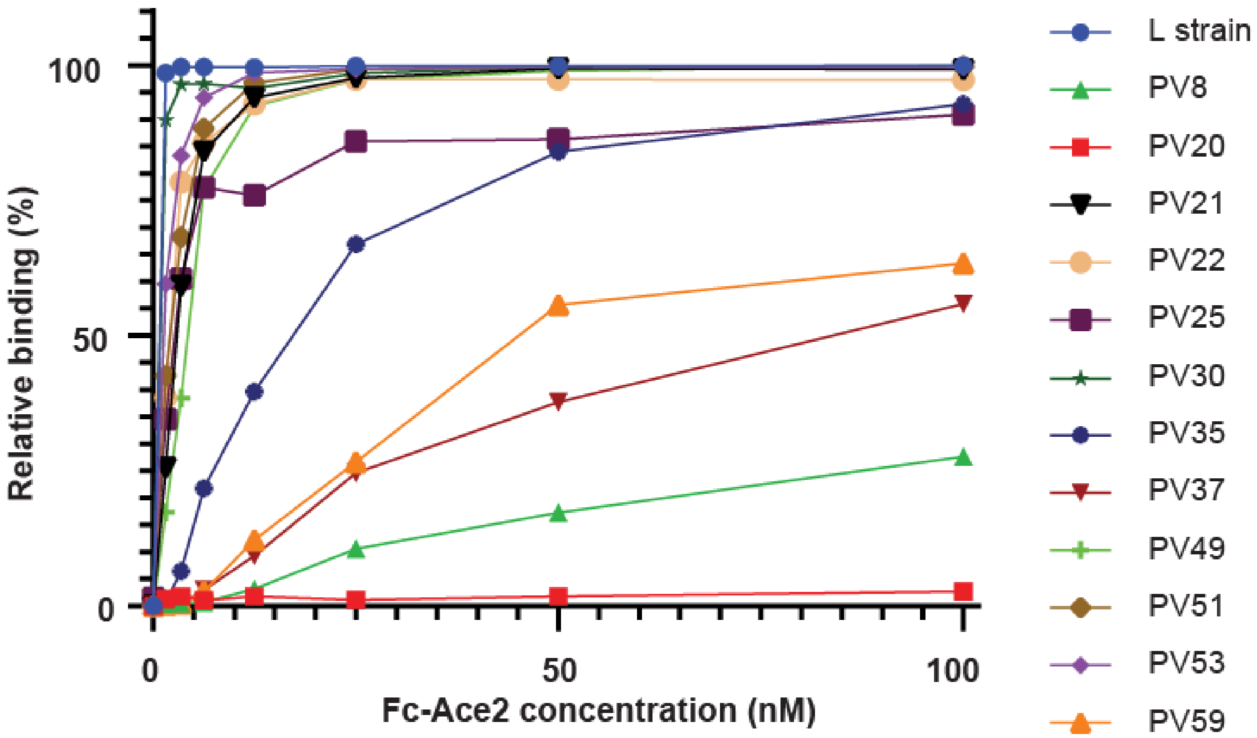

Figure S6. Dose response curves of yeast cells displaying the indicated RBD potential variants (PV) and Fc-ACE2 at decreasing concentrations. Relative binding is shown as the % of RBD-expressing cells.

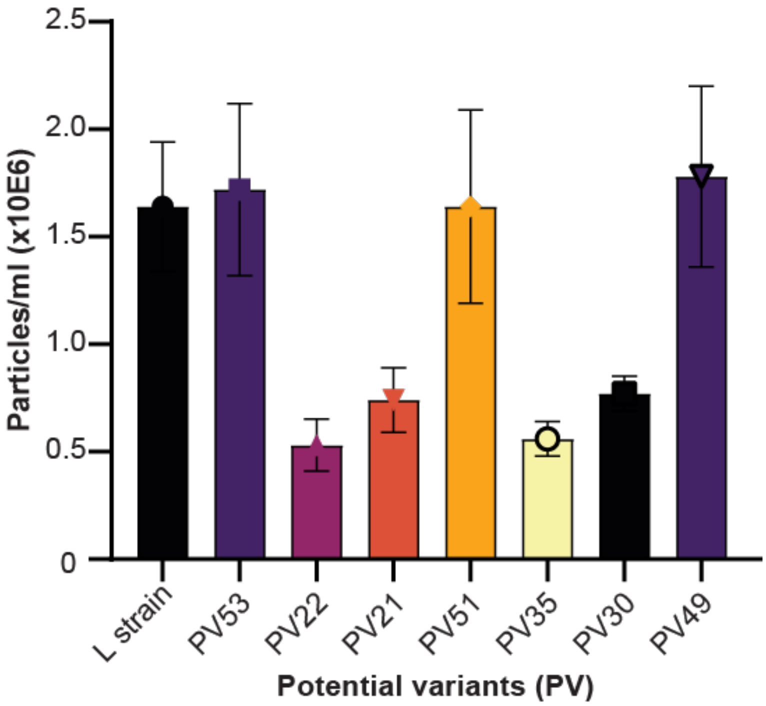

Figure S7. **Quantification of pseudotyped lentiviral particles by real time-PCR**. The viral titers were quantified by real time PCR and normalized to use equal amounts of all RBD variants in the transduction experiments. The obtained viral titers are consistent with these from Cronshaw et al. 2020.

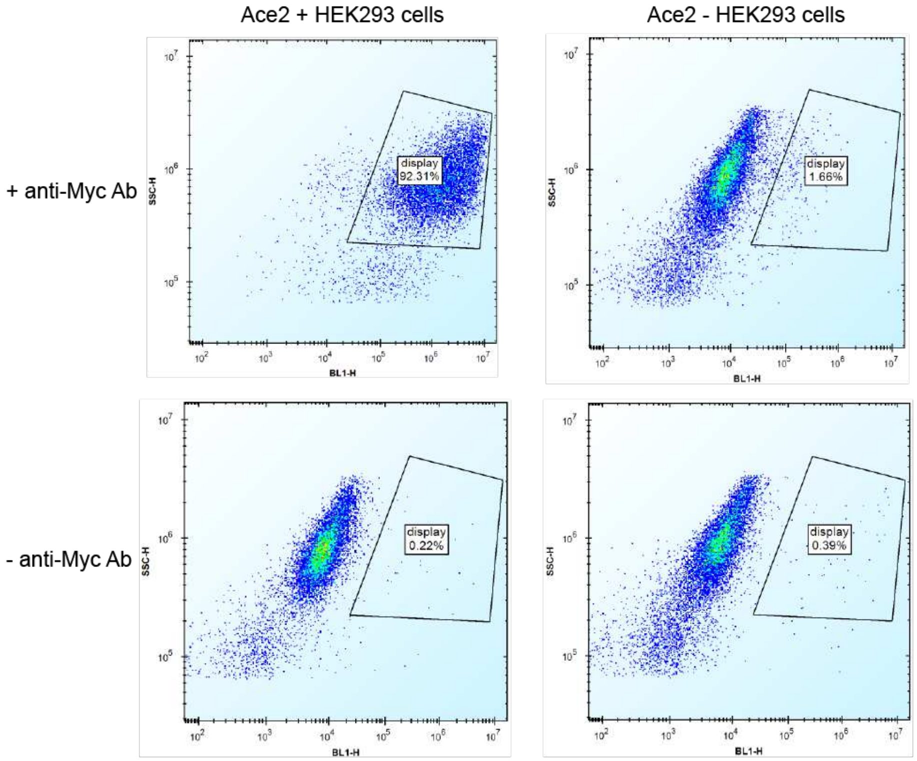

Figure S8. Validation of the Ace2+ transient cell line. To obtain the ACE2+ transient cell line, HEK293 cells were transiently transfected with a mammalian expression plasmid encoding for human ACE2 with an N-terminal Myc tag (Addgene #141185). Thus, ACE2 expression could be validated by staining HEK293 with a FITC-conjugated anti-Myc antibody.

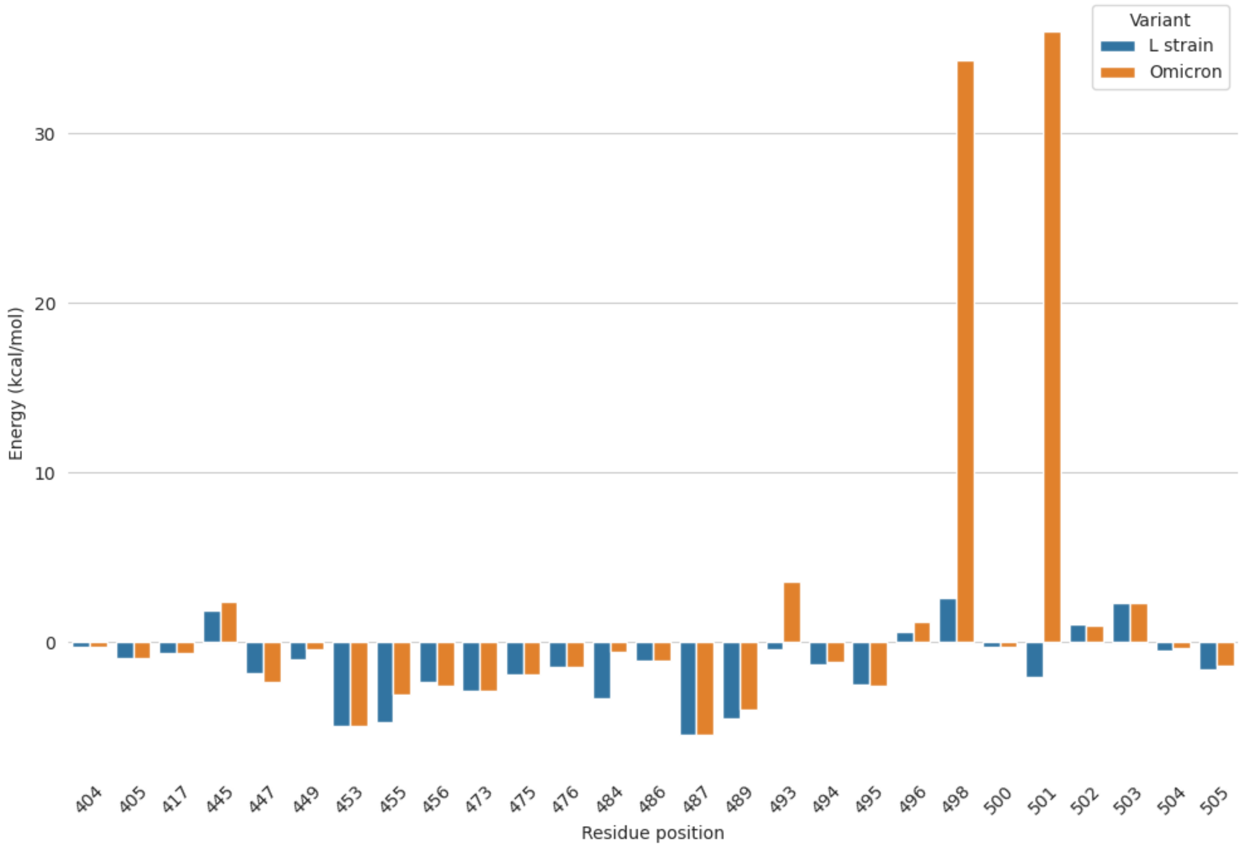

Figure S9. Per residue score breakdown of the L strain and omicron RBD/ACE2 complex form. Scoring was done on the initial backbone of the L strain RBD/ACE2 complex. Mutable interface residues are shown.

	PV1	PV2	PV3	PV4	PV5	PV6	PV7	PV8	PV9	PV10	PV11	PV12	PV13	PV14	PV15	PV16	PV17	PV18	PV19	PV20	PV21	PV22	PV23	PV24	PV25	PV26	PV27	PV28	PV29	PV30	PV31	PV32	PV33	PV34	PV35	PV36	PV37	PV38	PV39	PV40	PV41	PV42	PV43	PV44	PV45	PV46	PV47	PV48	PV49	PV50	PV51	PV52	PV53	PV54	PV55	PV56	PV57	PV58	PV59
PV1	0	7	9	11	7	9	12	9	8	10	8	10	8	7	10	7	7	10	9	10	8	11	8	8	11	8	10	8	6	12	6	10	7	9	10	10	8	11	9	6	9	8	8	11	6	9	9	10	10	10	7	8	11	13	12	10	8	6	8
PV2	7	0	7	12	8	11	13	11	11	12	8	10	7	7	11	6	8	8	8	8	8	12	9	7	14	9	10	10	8	10	7	12	6	10	13	9	9	9	9	11	10	11	8	13	8	8	10	8	11	12	7	11	9	12	10	12	7	9	11
PV3	9	7	0	11	12	7	12	12	11	13	6	12	10	8	10	7	6	12	9	8	5	11	9	10	13	8	9	10	6	10	8	11	8	10	11	9	10	5	10	12	10	9	7	12	6	5	9	8	8	11	6	13	6	12	12	11	6	8	11
PV4	11	12	11	0	9	11	7	10	8	7	9	7	8	8	9	11	8	7	7	10	13	10	9	8	12	10	9	9	9	10	13	5	11	11	10	9	10	12	11	12	9	12	9	8	14	11	9	8	9	7	10	12	12	9	7	9	11	8	6
PV5	7	8	12	9	0	10	11	10	7	11	7	7	5	7	11	9	10	11	11	12	11	11	9	9	11	9	12	11	8	11	7	10	8	9	11	12	10	14	9	9	8	9	10	11	10	11	11	13	13	11	10	6	13	11	11	12	11	10	9
PV6	9	11	7	11	10	0	12	10	9	10	9	11	9	8	6	9	6	13	9	9	7	9	8	11	8	6	8	8	7	8	6	7	9	10	10	11	12	9	9	12	8	10	7	8	9	7	9	13	9	12	8	10	9	9	12	11	11	7	10
PV7	12	13	12	7	11	12	0	10	13	6	9	9	9	9	9	14	12	10	7	13	14	10	10	10	8	8	9	10	12	9	12	9	12	12	7	7	11	11	10	10	9	12	10	9	11	9	6	8	7	9	9	11	10	7	9	9	12	10	10
PV8	9	11	12	10	10	10	10	0	7	9	11	8	11	12	7	8	11	9	9	8	7	7	10	12	8	11	7	11	8	6	11	8	7	8	7	9	11	9	7	8	13	12	9	7	11	12	10	10	12	7	13	7	8	10	6	11	10	9	7
PV9	8	11	11	8	7	9	13	7	0	11	8	10	10	10	8	8	8	9	9	9	9	8	9	9	10	10	9	9	8	10	11	8	8	6	9	11	9	12	11	8	12	7	9	8	11	11	11	12	12	7	11	6	11	12	11	10	10	8	7
PV10	10	12	13	7	11	10	6	9	11	0	13	9	9	9	7	12	11	9	7	11	14	9	9	9	7	8	8	8	10	9	11	6	11	10	7	6	9	11	7	9	9	12	8	7	11	10	7	6	8	9	10	9	11	7	6	8	10	9	7
PV11	8	8	6	9	7	9	9	11	8	13	0	11	9	6	10	6	8	12	9	9	6	9	10	11	12	8	9	11	9	11	9	11	6	7	9	10	9	9	12	11	9	9	7	12	7	7	10	11	11	10	8	10	10	14	14	11	10	8	10
PV12	10	10	12	7	7	11	9	8	10	9	11	0	7	7	9	10	11	9	10	8	11	11	10	8	13	8	11	7	8	8	12	9	13	12	13	10	12	9	6	12	8	11	10	8	13	11	9	11	11	13	10	10	9	11	8	14	7	10	10
PV13	8	7	10	8	5	9	9	11	10	9	9	7	0	6	9	10	8	10	9	10	12	13	7	6	13	7	11	8	7	9	7	9	8	12	13	9	12	12	7	12	6	12	7	9	11	8	8	11	11	12	7	9	12	8	9	13	10	7	10
PV14	7	7	8	8	7	8	9	12	10	9	6	7	6	0	10	7	8	11	7	7	9	13	8	8	13	8	7	8	8	11	10	9	8	9	13	7	9	9	8	11	7	11	4	9	8	6	9	9	8	13	6	10	9	12	12	13	7	7	11
PV15	10	11	10	9	11	6	9	7	8	7	10	9	9	10	0	10	7	11	7	9	11	9	7	7	9	5	6	6	8	8	10	7	7	7	8	8	12	9	9	10	9	10	7	5	11	9	8	9	12	9	10	8	11	9	9	11	8	6	9
PV16	7	6	7	11	9	9	14	8	8	12	6	10	10	7	10	0	8	9	9	6	5	9	9	11	13	9	7	11	7	10	10	9	6	7	11	10	9	8	9	11	11	11	6	11	8	9	12	10	12	11	9	10	9	15	11	13	8	8	9
PV17	7	8	6	8	10	6	12	11	8	11	8	11	8	8	7	8	0	10	8	9	9	12	4	6	13	7	8	7	6	11	8	8	8	10	11	8	9	9	8	9	9	8	7	10	9	8	9	10	9	7	5	11	11	12	11	9	9	6	9
PV18	10	8	12	7	11	13	10	9	9	9	12	9	10	11	11	9	10	0	7	10	12	8	8	9	11	9	10	10	10	11	12	7	12	12	11	10	8	13	10	11	12	11	12	11	13	12	10	8	10	8	11	10	10	9	9	9	10	11	8
PV19	9	8	9	7	11	9	7	9	9	7	9	10	9	7	7	9	8	7	0	8	10	9	8	8	8	7	5	7	10	9	11	6	8	8	9	5	6	11	11	9	10	11	8	9	10	7	7	5	6	8	8	10	6	8	10	9	7	8	8
PV20	10	8	8	10	12	9	13	8	9	11	9	8	10	7	9	6	9	10	8	0	6	9	11	10	14	10	8	9	8	7	13	10	8	8	13	9	10	6	8	13	11	13	6	9	11	8	9	10	11	13	9	8	7	12	9	15	7	7	11
PV21	8	8	5	13	11	7	14	7	9	14	6	11	12	9	11	5	9	12	10	6	0	8	11	13	11	10	10	11	9	8	8	12	6	8	11	11	10	5	9	12	12	9	8	12	6	6	12	12	10	12	9	11	5	13	12	12	9	9	11
PV22	11	12	11	10	11	9	10	7	8	9	9	11	13	13	9	9	12	8	9	9	8	0	14	13	7	9	11	11	10	6	11	8	11	9	5	13	8	11	12	11	12	10	12	10	12	12	9	11	10	9	13	9	9	10	9	10	12	11	7
PV23	8	9	9	9	9	8	10	10	9	9	10	10	7	8	7	9	4	8	8	11	11	14	0	8	11	6	7	7	8	13	9	8	8	10	12	6	10	10	5	8	9	10	7	9	8	7	10	9	11	7	6	8	11	9	11	9	10	7	11
PV24	8	7	10	8	9	11	10	12	9	9	11	8	6	8	7	11	6	9	8	10	13	13	8	0	13	7	10	6	9	9	8	10	9	11	12	8	10	10	10	10	9	6	9	9	11	9	7	9	8	10	7	11	11	9	9	9	6	8	10
PV25	11	14	13	12	11	8	8	8	10	7	12	13	13	13	9	13	13	11	8	14	11	7	11	13	0	10	10	10	13	10	8	10	11	9	4	9	8	12	11	7	12	9	12	10	9	10	9	10	8	7	12	8	9	6	11	5	13	12	9
PV26	8	9	8	10	9	6	8	11	10	8	8	8	7	8	5	9	7	9	7	10	10	9	6	7	10	0	8	6	8	8	7	7	10	10	9	9	11	9	9	11	6	8	9	9	9	7	5	10	9	12	7	9	9	10	12	11	8	8	10
PV27	10	10	9	9	12	8	9	7	9	8	9	11	11	7	6	7	8	10	5	8	10	11	7	10	10	8	0	10	9	9	13	5	7	7	8	5	9	8	10	8	12	12	4	7	9	9	9	7	8	8	10	10	8	11	9	11	8	8	9
PV28	8	10	10	9	11	8	10	11	9	8	11	7	8	8	6	11	7	10	7	9	11	11	7	6	10	6	10	0	10	9	9	10	10	10	12	6	9	10	8	8	8	9	9	8	11	7	7	10	9	10	6	10	9	9	12	11	8	6	11
PV29	6	8	6	9	8	7	12	8	8	10	9	8	7	8	8	7	6	10	10	8	9	10	8	9	13	8	9	10	0	9	9	7	9	10	10	11	11	9	7	11	9	11	7	8	9	10	9	9	11	10	8	9	10	12	8	13	7	7	7
PV30	12	10	10	10	11	8	9	6	10	9	11	8	9	11	8	10	11	11	9	7	8	6	13	9	10	8	9	9	9	0	10	9	10	12	8	10	13	8	9	12	10	12	10	9	13	10	6	12	9	12	11	12	6	9	6	14	10	9	10
PV31	6	7	8	13	7	6	12	11	11	11	9	12	7	10	10	10	8	12	11	13	8	11	9	8	8	7	13	9	9	10	0	12	7	12	10	11	10	11	10	10	8	8	11	13	7	8	8	12	10	11	7	10	11	8	12	8	11	10	10
PV32	10	12	11	5	10	7	9	8	8	6	11	9	9	9	7	9	8	7	6	10	12	8	8	10	10	7	5	10	7	9	12	0	11	10	8	10	9	12	10	11	11	12	8	6	13	12	9	8	8	8	11	10	11	9	6	10	10	9	4
PV33	7	6	8	11	8	9	12	7	8	11	6	13	8	8	7	6	8	12	8	8	6	11	8	9	11	10	7	10	9	10	7	11	0	5	10	8	10	9	10	10	11	12	5	9	7	7	12	9	13	9	9	8	11	10	10	11	10	6	10
PV34	9	10	10	11	9	10	12	8	6	10	7	12	12	9	7	7	10	12	8	8	8	9	10	11	9	10	7	10	10	12	12	10	5	0	8	9	8	8	11	8	13	9	6	6	8	9	13	9	12	8	11	6	11	12	11	10	9	8	9
PV35	10	13	11	10	11	10	7	7	9	7	9	13	13	13	8	11	11	11	9	13	11	5	12	12	4	9	8	12	10	8	10	8	10	8	0	10	8	10	12	7	12	9	10	9	9	12	7	9	8	5	12	9	11	10	8	7	12	11	6
PV36	10	9	9	9	12	11	7	9	11	6	10	10	9	7	8	10	8	10	5	9	11	13	6	8	9	9	5	6	11	10	11	10	8	9	10	0	7	8	6	6	10	11	6	9	8	6	7	6	7	7	6	10	7	8	10	9	8	8	11
PV37	8	9	10	10	10	12	11	11	9	9	9	12	12	9	12	9	9	8	6	10	10	8	10	10	8	11	9	9	11	13	10	9	10	8	8	7	0	11	11	6	13	8	10	12	8	10	10	7	7	6	8	9	9	10	11	7	8	12	7
PV38	11	9	5	12	14	9	11	9	12	11	9	9	12	9	9	8	9	13	11	6	5	11	10	10	12	9	8	10	9	8	11	12	9	8	10	8	11	0	7	11	12	9	6	9	6	6	10	9	8	11	8	12	6	11	8	11	7	9	12
PV39	9	9	10	11	9	9	10	7	11	7	12	6	7	8	9	9	8	10	11	8	9	12	5	10	11	9	10	8	7	9	10	10	10	11	12	6	11	7	0	9	9	12	7	8	9	8	10	10	11	10	7	8	9	9	7	12	9	8	11
PV40	6	11	12	12	9	12	10	8	8	9	11	12	12	11	10	11	9	11	9	13	12	11	8	10	7	11	8	8	11	12	10	11	10	8	7	6	6	11	9	0	13	7	10	11	8	11	9	9	9	5	9	7	10	11	11	7	10	9	9
PV41	9	10	10	9	8	8	9	13	12	9	9	8	6	7	9	11	9	12	10	11	12	12	9	9	12	6	12	8	9	10	8	11	11	13	12	10	13	12	9	13	0	12	9	11	11	8	7	12	11	14	7	11	12	11	13	13	11	8	12
PV42	8	11	9	12	9	10	12	12	7	12	9	11	12	11	10	11	8	11	11	13	9	10	10	6	9	8	12	9	11	12	8	12	12	9	9	11	8	9	12	7	12	0	12	12	7	9	9	12	7	9	9	9	9	12	14	6	8	12	10
PV43	8	8	7	9	10	7	10	9	9	8	7	10	7	4	7	6	7	12	8	6	8	12	7	9	12	9	4	9	7	10	11	8	5	6	10	6	10	6	7	10	9	12	0	6	7	6	10	8	9	10	7	9	10	11	9	12	8	5	10
PV44	11	13	12	8	11	8	9	7	8	7	12	8	9	9	5	11	10	11	9	9	12	10	9	9	10	9	7	8	8	9	13	6	9	6	9	9	12	9	8	11	11	12	6	0	12	10	10	10	10	9	11	8	12	8	7	12	10	7	8
PV45	6	8	6	14	10	9	11	11	11	11	7	13	11	8	11	8	9	13	10	11	6	12	8	11	9	9	9	11	9	13	7	13	7	8	9	8	8	6	9	8	11	7	7	12	0	5	10	9	8	10	7	9	8	11	13	8	9	10	10
PV46	9	8	5	11	11	7	9	12	11	10	7	11	8	6	9	9	8	12	7	8	6	12	7	9	10	7	9	7	10	10	8	12	7	9	12	6	10	6	8	11	8	9	6	10	5	0	8	9	7	12	5	10	6	8	13	10	9	6	13
PV47	9	10	9	9	11	9	6	10	11	7	10	9	8	9	8	12	9	10	7	9	12	9	10	7	9	5	9	7	9	6	8	9	12	13	7	7	10	10	10	9	7	9	10	10	10	8	0	9	6	10	7	9	8	9	10	10	9	9	9
PV48	10	8	8	8	13	13	8	10	12	6	11	11	11	9	9	10	10	8	5	10	12	11	9	9	10	10	7	10	9	12	12	8	9	9	9	6	7	9	10	9	12	12	8	10	9	9	9	0	8	8	9	11	8	9	7	7	6	10	8
PV49	10	11	8	9	13	9	7	12	12	8	11	11	11	8	12	12	9	10	6	11	10	10	11	8	8	9	8	9	11	9	10	8	13	12	8	7	7	8	11	9	11	7	9	10	8	7	6	8	0	9	7	13	6	8	10	7	9	11	9
PV50	10	12	11	7	11	12	9	7	7	9	10	13	12	13	9	11	7	8	8	13	12	9	7	10	7	12	8	10	10	12	11	8	9	8	5	7	6	11	10	5	14	9	10	9	10	12	10	8	9	0	10	9	12	9	8	5	12	10	6
PV51	7	7	6	10	10	8	9	13	11	10	8	10	7	6	10	9	5	11	8	9	9	13	6	7	12	7	10	6	8	11	7	11	9	11	12	6	8	8	7	9	7	9	7	11	7	5	7	9	7	10	0	11	9	10	12	10	8	7	11
PV52	8	11	13	12	6	10	11	7	6	9	10	10	9	10	8	10	11	10	10	8	11	9	8	11	8	9	10	10	9	12	10	10	8	6	9	10	9	12	8	7	11	9	9	8	9	10	9	11	13	9	11	0	12	9	11	10	11	9	9
PV53	11	9	6	12	13	9	10	8	11	11	10	9	12	9	11	9	11	10	6	7	5	9	11	11	9	9	8	9	10	6	11	11	11	11	11	7	9	6	9	10	12	9	10	12	8	6	8	8	6	12	9	12	0	10	11	11	6	11	12
PV54	13	12	12	9	11	9	7	10	12	7	14	11	8	12	9	15	12	9	8	12	13	10	9	9	6	10	11	9	12	9	8	9	10	12	10	8	10	11	9	11	11	12	11	8	11	8	9	9	8	9	10	9	10	0	7	7	13	10	10
PV55	12	10	12	7	11	12	9	6	11	6	14	8	9	12	9	11	11	9	10	9	12	9	11	9	11	12	9	12	8	6	12	6	10	11	8	10	11	8	7	11	13	14	9	7	13	13	10	7	10	8	12	11	11	7	0	10	10	10	6
PV56	10	12	11	9	12	11	9	11	10	8	11	14	13	13	11	13	9	9	9	15	12	10	9	9	5	11	11	11	13	14	8	10	11	10	7	9	7	11	12	7	13	6	12	12	8	10	10	7	7	5	10	10	11	7	10	0	12	12	8
PV57	8	7	6	11	11	11	12	10	10	10	10	7	10	7	8	8	9	10	7	7	9	12	10	6	13	8	8	8	7	10	11	10	10	9	12	8	8	7	9	10	11	8	8	10	9	9	9	6	9	12	8	11	6	13	10	12	0	10	10
PV58	6	9	8	8	10	7	10	9	8	9	8	10	7	7	6	8	6	11	8	7	9	11	7	8	12	8	8	6	7	9	10	9	6	8	11	8	12	9	8	9	8	12	5	7	10	6	9	10	11	10	7	9	11	10	10	12	10	0	10
PV59	8	11	11	6	9	10	10	7	7	7	10	10	10	11	9	9	9	8	8	11	11	7	11	10	9	10	9	11	7	10	10	4	10	9	6	11	7	12	11	9	12	10	10	8	10	13	9	8	9	6	11	9	12	10	6	8	10	10	0

Table S1. Distance matrix of the 59 PVs. Pairwise Hamming distances between all potential variants.

		With local minimas (3,272)		With all predicted RBDs (4,507,187)	
**Variant**	**RBD (27 AAs)**	**Min. # of mutations**	**# of matches**	**Min. # of mutations**	**# of matches**
Beta, V2 (B.1.351)	GDNVGYYLFYAGKFNYQSYGQT YGVGY	6	2	3	1
Alpha, V1 (B.1.1.7)	GDKVGYYLFYAGEFNYQSYGQT YGVGY	5	2	2	4
Gamma, V3 (P.1)	GDTVGYYLFYAGKFNYQSYGQTY GVGY	6	2	4	40
Delta (B.1.617.2)	**GDKVGYYLFYAGEFNYQSYGQT NGVGY**	4	2	1	2
Kappa (B.1.617.1)	GDKVGYYLFYAGQFNYQSYGQT NGVGY	5	5	2	8
Eta (B.1.525)	GDKVGYYLFYAGKFNYQSYGQT NGVGY	5	2	2	2
Iota (B.1.526)	GDKVGYYLFYAGKFNYQSYGQT NGVGY	5	2	2	2
Lambda (C.37)	GDKVGYYLFYAGEFNYQSYGQT NGVGY	4	2	1	2
Mu (B.1.621)	GDKVGYYLFYAGKFNYQSYGQT YGVGY	6	2	3	4
Omicron (BA.1)	GDNVGYYLFYAGAFNYRSYSRTY GVGH	>8	None (<=8)	7	21
Omicron (BA.2)	GNNVGYYLFYAGAFNYRSYGRTY GVGH	>8	None (<=8)	6	2
L strain (WT)	GDKVGYYLFYAGEFNYQSYGQT NGVGY	4	2	1	2

Table S2. Distances between variants of concern, local minima and potential variants in the filtered fitness landscape.

Potential Variant (PV)	Mutations on the path	Distance from wild type	Path length
PV49	V503E,V445F,S494A,Y505W,T500A,N501A,K417W	7	7
PV30	V503E,S494P,V445E,E484Q,K417L,Y453F,Y473F	7	7
PV53	V503E,T500A,V445L,L445W,Y453F,S494P,K417L,N501A,Q493V	8	9
PV21	V503E,D405G,G504E,K417R,V445L,T500A,L445W,S494P,Q498V,L455M,Q493L,Y473F,Y453F,R417L,N501A,V498Q	12	16
PV22	V503E,D405G,S494P,K417E,G405R,V445E,Y505W,L455K,Q493L,R405S,Y453F,E417V,Y473F	10	13
PV51	V503E,D405G,G504E,T500A,S494A,V445D,Q498H,K417E,N501T	9	9
PV25	V503E,S494P,V445E,Y495F,K417E,Y505W,L455K,Q493L,Q498T,N501A,E417A	10	11

Table S3. Most probable mutational paths from L strain to antibody escaping PVs. For each PV, we show all mutations on the most probable path in the order in which they appear as well as the Hamming distance from the L strain and the path length.

Mismatch pos.	Potential PV in the filtered fitness landscape	# of hits in GISAID	Comment
494	GDKVGYYLFYAGEFNYQAYGQTNGDGY	7	
498	GDKVGYYLFYAGEFNYQSYGVTNGLGY	8	
498	GDKVGYYLFYAGEFNYQSYGVTNGDGY	7	
498	GDKVGYYLFYAGEFNYQSYGVTNGAGY	48	
498	GDKDGYYLFYAGEFNYQSYGVTNGVGY	7	
405	GGKVGYYLFYAGEFNYQSYGQTNGVEY	1	
445	GDKEGYYLFYAGEFNYQSYGQTNGAGY	48	
445	GDKHGYYLFYAGEFNYQSYGQTNGAGY	48	
445	GDKEGYYLFYAGEFNYQSYGQTNGDGY	7	
445	GDKHGYYLFYAGEFNYQSYGQTNGDGY	7	
445	GDKMGYYLFYAGEFNYQSYGQTNGDGY	7	
445	GDKEGYYLFYAGEFNYQSYGQTNGLGY	8	
445	GDEEGYYLFYAGEFNYQSYGQTNGVGY	29	
503	GAKVGYYLFYAGEFNYQSYGQTNGEGY	14	
503	GDAVGYYLFYAGEFNYQSYGQTNGEGY	1	
503	GDEVGYYLFYAGEFNYQSYGQTNGEGY	29	
503	GDKAGYYLFYAGEFNYQSYGQTNGEGY	187	
503	GDKDGYYLFYAGEFNYQSYGQTNGEGY	7	
503	GDKDGYYLFYAGEFNYQSYGQTNGH	7	

	GY		
503	GDKFGYYLFYAGEFNYQSYGQTNGEGY	218	
503	GDKFGYYLFYAGEFNYQSYGQTNGSGY	218	
503	GDKGGYYLFYAGEFNYQSYGQTNGEGY	6	
503	GDKIGYYLFYAGEFNYQSYGQTNGEGY	211	
503	GDKIGYYLFYAGEFNYQSYGQTTGEGY	1	
503	GDKLGYYLFYAGEFNYQSYGQTNGEGY	2	
503	GDKSGYYLFYAGEFNYQSYGQTNGEGY	1	
503	GDKVGFYLFYAGEFNYQSYGQTNGEGY	5	
503	GDKVGYYLFFAGEFNYQSYGQTNGEGY	44	
503	GDKVGYYLFYAGEFNYQAYGQTNGDGY	148	
503	GDKVGYYLFYAGEFNYQAYGQTNGEGY	148	
503	GDKVGYYLFYAGEFNYQAYGQTNGHGY	148	
503	GDKVGYYLFYAGEFNYQAYGQTNGSGY	148	
503	GDKVGYYLFYAGEFNYQPFGQTNGEGY	1	
503	GDKVGYYLFYAGEFNYQPYGQTNGEGY	7 268	
503	GDKVGYYLFYAGEFNYQPYGQTNGSGY	7 268	
503	GDKVGYYLFYAGEFNYQPYGQTTGEGY	6	
503	GDKVGYYLFYAGEFNYQSFGQTNGEGY	1	
503	GDKVGYYLFYAGEFNYQSYGQANGEGY	14	
503	GDKVGYYLFYAGEFNYQSYGQTNGEGW	18	

503	GDKVGYYLFYAGEFNYQSYGQTNGEGY	4 884 812	L/Delta/Lambda
503	GDKVGYYLFYAGEFNYQSYGQTNGSGY	4 884 812	L/Delta/Lambda
503	GDKVGYYLFYAGEFNYQSYGQTTGEGY	4 593	
503	GDKYGYYLFYAGEFNYQSYGQTNGEGY	1	
503	GDMVGYYLFYAGEFNYQSYGQTNGEGY	16	
503	GDNVGYYLFYAGEFNYQPYGQTNGEGY	2	
503	GDNVGYYLFYAGEFNYQSYGQTNGEGY	7 817	
503	GDQVGYYLFYAGEFNYQSYGQTNGEGY	4	
503	GDRVGYFLFYAGEFNYQSYGQTNGEGY	2	
503	GDRVGYYLFYAGEFNYQSYGQTNGEGY	70	
503	GDSVGYYLFYAGEFNYQSYGQTNGEGY	1	
503	GGKVGYYLFYAGEFNYQSYGQTNGEGY	96	
	Total unique hits at distance 1	4 905 597	

Table S4. Number of GISAID RBD sequences that would fit in the filtered fitness landscape. List of all predicted RBDs from the filtered sequence landscape having a match within at most one mutation in the spike protein GISAID database, filtered for complete sequences. A total of 4,905,597 GISAID sequences would fit in our filtered sequence landscape, representing 67.7% of all GISAID sequences.

Variant	Sequence	Changes in ACE2 affinity according to DMS
PV21	GGLWGYFMFFAGEFNYLPYGQAAGEEY	-87
PV22	GSVEGYFKFFAGEFNYLPYGQTNGEGW	nd
PV30	GDLEGYFLFFAGQFNYQPYGQTNGEGY	-3.7
PV35	GDAEGYYKFFAGEFNYLAFGQTNGEGW	nd
PV49	GDWFGYYLFYAGEFNYQAYGQAAGEGW	-11.7
PV51	GGEDGYYLFYAGEFNYQAYGHATGEEY	-162.18
PV53	GDLWGYFLFYAGEFNYVPYGQAAGEGY	-7.24

Table S5. Deep Mutational Scanning (DMS) misses some of the mutations contained in the identified PVs, since it does not consider epistatic effects. DMS identified the single-point mutations contained in some of our infective PVs (e.g. PV30, PV53). However, the mutations found in e.g. PV21 and PV51 are highly detrimental to ACE2 binding according to DMS, while we showed that these PVs are as infectious as the L strain. Single mutation effects were obtained from Bloom lab's github repository: https://github.com/jbloomlab/SARS-CoV-2-RBD_DMS, and each PV was scored by summing up the values of each individual mutation. A negative value means weaker predicted binding for human ACE2. PV22 and PV35 could not be scored because at least one of their mutations was not observed in the DMS libraries.
