# Supplementary material for: Deep Evolutionary Forecasting identifies highly-mutated SARS-CoV-2 variants via functional sequence-landscape enumeration
Source: Res Sq. 2022 Dec 2:rs.3.rs-2248327. Preprint. [Version 1] doi: 10.21203/rs.3.rs-2248327/v1 (PMC9727770; doi:10.21203/rs.3.rs-2248327/v1)
Supplement: Supplementary file 1 [file NIHPPRS2248327v1-supplement-1.pdf]

Supplementary Files

This is a list of supplementary files associated with this preprint. Click to download.

- SUPPLEMENTARYINFORMATION.docx
